# A Comparison of White Matter Brain Differences in Monolingual and Highly Proficient Multilingual Speakers

**DOI:** 10.1162/nol_a_00144

**Published:** 2024-06-03

**Authors:** Ludmila Midrigan-Ciochina, Kayla P. Vodacek, Cristina Sewell, David P. Corina

**Affiliations:** Center for Mind and Brain, University of California, Davis, Davis, CA, USA; Department of Linguistics and Human Ecology, University of California, Davis, Davis, CA, USA; Department of Linguistics and Psychology, University of California, Davis, Davis, CA, USA

**Keywords:** high language proficiency, morphometry, multilingualism, white matter

## Abstract

Language processing relies on the communication between brain regions that is achieved through several white matter tracts, part of the dorsal, ventral, and medial pathways involved in language processing and control (Coggins et al., 2004; Friederici & Gierhan, 2013; Hickok & Poeppel, 2007; Luk et al., 2011). While changes in white matter tract morphology have been reported as a function of second language learning in bilinguals, little is known about changes that may be present in multilanguage users. Here we investigate white matter morphometry in a group of highly proficient multilinguals, (individuals with proficiency in four or more languages), compared to a group of monolinguals. White matter morphometry was quantified using a fixel-based analysis (Raffelt et al., 2015; Raffelt et al., 2017; Tournier et al., 2007). Higher fiber cross-section and lower fiber density values were observed for the multilinguals, in the dorsal pathways (superior longitudinal fasciculus and arcuate fasciculus) and the ventral pathway, including the inferior fronto-occipital fasciculus, inferior longitudinal fasciculus, and the uncinate fasciculus. Segments of the corpus callosum, the fornix, and the cortico-spinal tract showed decreases in all three morphometry measures for multilinguals. The findings suggest differential efficiencies in neural communication between domain-specific language regions and domain-general cognitive processes underlying multilingual language use. We discuss the results in relation to the bilingual Anterior to Posterior and Subcortical Shift (BAPSS) hypothesis (Grundy et al., 2017) and the Dynamic Restructuring Model (Pliatsikas, 2020).

## INTRODUCTION

Language processing relies on the communication between brain regions that is achieved through several white matter (WM) tracts (Friederici, 2009), which include dorsal and ventral language pathways (Friederici & Gierhan, 2013; Hickok & Poeppel, 2007), and a medial pathway involved in language control (Coggins et al., 2004; Luk et al., 2011). In recent years, there has been an interest in quantifying and contrasting the WM tracts that show restructuring in response to the task of learning and using second languages in bi/multilinguals (Pliatsikas, 2020; Pliatsikas et al., 2015). However, the available literature on second languages stems from studies comparing bilinguals, that is, speakers of one second language, to monolinguals. To our knowledge, there are no studies that have looked at highly proficient lifelong multilinguals compared to baseline monolinguals. Moreover, there are only very few studies that have investigated brain restructuring patterns in different groups of multilinguals (e.g., sequential vs. simultaneous), and the results from these remain mixed (e.g., Hämäläinen et al., 2017).

Multilingualism may differ in substantive ways from bilingualism The experience of practicing multiple versus two languages is different, therefore the brain changes associated with these experiences may be different. At a behavioral level, in any interactional context, the demands for interference control from a second language may vary greatly for multilinguals compared to bilinguals. In a single-language context, a speaker of three or more languages may need to control for interference from more than one language, compared to controlling for only one, if the speaker is bilingual. In a multilanguage context, a bilingual may only switch to one of the two languages, while a multilingual would need to juggle multiple languages at the same time. Evidence of brain changes associated with multilingualism compared to those of bilingualism is mixed. While bilingual evidence suggests generally greater functional activation and an increase followed by a normalization process of gray matter for bilinguals compared to monolinguals (Pliatsikas, 2019; Pliatsikas & Luk, 2016), recent studies point to different effects when looking at groups of multilinguals compared to monolinguals (Jouravlev et al., 2021; Midrigan-Ciochina et al., 2023). Increased efficiency in brain language networks related to multilinguals may lead to more extreme brain restructuring than observed in bilinguals, namely, reduction in functional activation due to more efficient language representation and processing, along with reductions in gray and white matter morphology, as a result of pruning (Giorgio et al., 2010).

Lastly, the traditional methods largely based on diffusion tensor imaging (DTI; Basser & Pierpaoli, 2011) suffer from several limitations and have been largely criticized in recent years (e.g., Farquharson & Tournier, 2016; Jones et al., 2013).

In an effort to address some of these literature gaps, in this study, we investigate WM morphometry in a group of highly immersed, highly proficient (in four or more languages) multilinguals, compared to a group of monolinguals. We make use of a recently developed method, namely, fixel-based analysis (Raffelt et al., 2015; Raffelt et al., 2017; Tournier et al., 2007), that uses a higher-order diffusion model (i.e., constrained spherical deconvolution [CSD], and can characterize specific fiber population orientations within voxels (e.g., Raffelt et al., 2011; Raffelt et al., 2012; Raffelt et al., 2017; Tournier et al., 2007; Tournier et al., 2011). This method provides a more sensitive and interpretable alternative to the traditional tensor models (Raffelt et al., 2015).

### Background Literature

The inherent individual variability among the bilingual participants (e.g., differences in language proficiency, number of languages known, age of acquisition, amount of immersion in a bilingual environment; Pliatsikas et al., 2020) foster models of trajectorial brain adaptations after various linguistic backgrounds. However, to date, the literature informing such models largely stems from longitudinal and training studies investigating comparisons between speakers of two languages (a.k.a. bilinguals) and monolinguals. From this literature a consistent pattern seems to surface, particularly, at an early stage of second language learning, bilinguals recruit more the anterior cortical brain areas while, with an increase in immersion and/or proficiency, a shift from the anterior cortical to the subcortical and posterior regions is observed (Anderson et al., 2018; Grundy et al., 2017; Pliatsikas, 2020). Along with these changes, we might expect parallel restructuring of WM. That is, initial diffusivity increases in anterior brain regions followed by decreases in posterior regions with increasing proficiency are predicted (e.g., the Dynamic Restructuring Model [DRM]; Pliatsikas, 2020), although these predictions are yet to be tested. These fluctuations in WM diffusivity are assumed to be the result of efficient connectivity, in response to high proficiency in second languages, when a second language skill has been firmly established.

The effects of patterns of brain restructuring associated with high proficiency in multiple languages and high immersion in multilanguage contexts, by means of long-time residence in a second language environment, remain an understudied topic. There are very few studies that have investigated connectivity patterns associated with high expertise in multiple languages. The few studies looking at participants that spoke more than two languages showed that simultaneous multilinguals (speakers of three languages that have acquired both second languages early in life) compared to sequential multilinguals (speakers that acquired a third language later in life, after two languages have been acquired) display increased fractional anisotropy (FA) in the left arcuate fasciculus, decreased mean diffusivity (MD) in the right posterior arcuate fasciculus, and increased MD in the bilateral inferior fronto occipital fasciculus (Hämäläinen et al., 2017). How these differences compare to baseline monolinguals, is still unknown, due to the lack of studies comparing multilinguals to monolinguals.

The available evidence, coming from WM changes in studies of high proficiency bilinguals provides mixed results as well (for a recent review see Pliatsikas, 2019; Pliatsikas et al., 2020). Some studies show increases in WM FA (the directional/preferential asymmetry of water diffusion along a specific axis) and axial diffusivity (AD; i.e., greater water displacement along rather than perpendicular to the principal axis) for bi/multilinguals compared to monolinguals (e.g., García-Pentón et al., 2014; Pliatsikas et al., 2015; Qi et al., 2015; Schlegel et al., 2012). These are generally accompanied by decreases in radial diffusivity (RD; diffusion perpendicular to the fiber) and MD (mean water displacement along all axes). Other bilingual studies show the opposite (e.g., Cummine & Boliek, 2013; Elmer et al., 2014; Gold et al., 2013; Kuhl et al., 2016), and/or mixed patterns of effects (Mohades et al., 2012; Singh et al., 2018), reporting decreased FA and increased RD, in bilinguals compared to monolinguals in several WM tracts of dorsal, ventral, and medial pathways. One trend that is observed in the background of the participants showing these patterns of results is potentially higher opportunities to engage in bilingual environments (e.g., simultaneous interpreters; Elmer et al., 2011) and/or longer experience with the second languages learned (e.g., simultaneous vs. sequential bilinguals, Mohades et al., 2012; and lifelong bilinguals, Gold et al., 2013).

Lastly, the changes in the WM diffusivity in bilingual literature have been largely estimated with the DTI model (Basser & Pierpaoli, 2011) and statistical analyses using region-of-interest (ROI) and voxel-based analysis (VBA; Ashburner & Friston, 2000; Schwarz et al., 2014). Despite the great advantages of the diffusion tensor model, it suffers from inherent limitations. Constructs obtained with tensor models (e.g., FA, MD, AD, and RD) are heavily confounded by issues including, and not limited to, partial volume effects (Papadakis et al., 2002), as well as biases in tract estimation due to crossing and multiple directions of fiber orientations (Jeurissen et al., 2013). The limitations of the tensor models have been largely discussed in recent years (e.g., Farquharson & Tournier, 2016; Jones et al., 2013; Tournier et al., 2011). For instance, in the bilingual literature, higher FA has been generally interpreted to indicate higher WM integrity (Luk et al., 2011), as a result of myelination (Pliatsikas, 2020), although prior efforts to characterize changes in WM (i.e., obtained with traditional diffusion tensor techniques) do not allow for the characterization of the independent contribution of myelination compared to the axonal membranes packing to measures of diffusivity, such as FA (Beaulieu, 2002).

In this study, to overcome some of the limitations of the traditionally used voxel-based analysis, we make use of a recently developed method, namely, fixel-based analysis (FBA; Raffelt et al., 2012; Raffelt et al., 2015; Raffelt et al., 2017; Tournier et al., 2007). This newer method uses a higher-order diffusion model (i.e., CSD; Tournier et al., 2007) and enables fiber tract-specific statistical analysis (e.g., Raffelt et al., 2012; Raffelt et al., 2017; Tournier et al., 2011). The local capacity of transfer information of a fiber bundle is inferred through measures of apparent fiber density (AFD). These include metrics of (a) intra-axonal volume of restricted water, manifested at the microstructural level (i.e., the fine structure of tissue, cellular level) as differences in fiber density (FD; Raffelt et al., 2012); (b) macrostructural volumetric changes of the bundle size (i.e., the gross structure of tissue, the whole bundle tissue) manifested as a difference in fiber cross-section (FC); and (c) a combination of both FD and FC (FDC; for a detailed description of these metrics, see Raffelt et al., 2015; Raffelt et al., 2017). FBAs not only account for crossing-fiber populations within voxels but also can give a more comprehensive, reliable, and biologically interpretable picture of ways in which axonal volume changes may manifest (Raffelt et al., 2017). Compared to other diffusion metrics, the FBA can account for both microstructural adaptations driven by FD changes, and captured by the FD metric, macrostructural changes, at the level of fiber-bundle cross-section, expressed in the FC metric, and total axonal-volume changes, expressed by the FDC metric. As with traditional DTI the functional significance of these more anatomically accurate fixel-based accounts continues to evolve (Dhollander et al., 2021).

### Current Study

We investigate WM morphometry in a group of highly immersed, highly proficient (in four or more languages) multilinguals, compared to a group of monolinguals. Due to the limited literature looking at brain changes associated with multilingualism, we develop our hypothesis based on predictions of recent models of brain restructuring patterns related to bilingualism, namely, the Bilingual Anterior to Posterior and Subcortical Shift (BAPSS) hypothesis (Grundy et al., 2017) and the DRM (Pliatsikas, 2020). The BPASS (Anderson et al., 2018; Grundy et al., 2017) suggests that with increased proficiency in a second language, bilinguals become more efficient by increasing important gray and white matter structures and facilitating communication between anterior and subcortical/posterior regions, often indexed through WM diffusion increases. Hence, we expect to observe increases in WM morphology in anterior segments of the dorsal and ventral pathways involved in language processing, namely, the inferior frontal-occipital fasciculus (IFOF), inferior longitudinal fasciculus (ILF), the uncinate fasciculus (UF), and the superior longitudinal fasciculus (SLF)/arcuate fasciculus (AF). In line with the predictions of the BAPSS, the DRM predicts further remodeling of brain areas related to high proficiency in an L2, often observed in white matter pathways involved in language control and interhemispheric communication. Namely, at the advanced stage of language expertise, the changes observed in earlier stages (i.e., differences in the anterior brain regions) start to fade as a result of greater efficiency within these areas (i.e., “remodeling” often manifested as cortical volume reductions (e.g., Pliatsikas, 2020), while further reductions in WM of the corpus callosum (CC) should be observed. These hypothesis stem from the DRM-specific prediction of decreases in the CC for peak efficiency second-language speakers (Pliatsikas, 2020). Decreases in CC anisotropy have been noted in most advanced bilingual speakers that are immersed in a bilingual environment and are exposed to complex bilingual contexts, such as simultaneous interpreters (Elmer et al., 2011, 2014). These patterns of decreased white and gray matter morphology in tracts and gray matter regions involved in language control have been reported in participants with higher proficiency (Singh et al., 2018) and/or longer history of second language use (Gold et al., 2013), as well as speakers that have high demands for language switching (e.g., simultaneous interpreters; e.g., Elmer et al., 2011). Therefore, we expected to see decreased morphology in the CC, a tract that is associated with language control, and interhemispheric communication.

We therefore, expect to observe lower WM morphology values within the CC. A list of specific predictions and expected directions of results is presented in Table 1.

**Table 1.** Predicted white matter restructuring patterns.

*Domain-specific pathways*
Greater white matter morphology values in the language pathways in the anterior brain regions with lower values in the posterior brain regions
- Increased FD, FC, and FDC in the *dorsal pathway* fiber bundles (SLF, AF)
- Increased FD, FC, and FDC in the *ventral pathway* fiber bundles (IFOF, ILF, UF)
*Domain-general pathways*
Decreases in the white matter morphology values in the corpus callosum
- Decreases in FD, FC, and FDC in the *medial pathway* fiber bundles (CC)

*Note*. FD = Fiber density, FC = fiber cross-section, FDC = fiber density and cross-section, SLF = superior longitudinal fasciculus, AF = arcuate fasciculus, IFOF = inferior fronto-occipital fasciculus, ILF = inferior longitudinal fasciculus, UF = unscinate fasciculus, CC = corpus collosum.

## MATERIALS AND METHODS

### Participants

The behavioral and magnetic resonance imaging (MRI) data were acquired from 30 right-handed participants, 15 native Romanian multilinguals (speakers of four or more languages), and 15 English monolinguals, following written informed consent, approved by the Institutional Review Board at the University of California, Davis. The participants reported no previous history of neurological or developmental disorders and were between 19 and 33 years of age, (*M* 24.8 yrs, *SD* 1.7 yrs for the monolingual group and *M* 27.6 yrs, *SD* 3.2 yrs for the multilinguals). Participants were consistent right-handers, as measured by the Edinburgh Handedness Inventory laterality index: mean of 85.8% for the monolinguals and a mean of 81.63% for the multilingual group, the scores ranging from 0 (no hand preference) to +100 (always using the right hand) for all participants (Oldfield, 1971). The multilingual participants were all born and raised in Moldova, a bilingual country with most of the population speaking Romanian and Russian fluently. Romanian was reported by all as a native language (L1); Russian (L2) was also learned starting elementary/middle school (*M* age of 5.8 yrs for the group) and was used every day in conversations and entertainment purposes (i.e., watching Russian television, reading books, listening to music, and singing Russian songs in church). All participants learned an L3 (e.g., French, German, or Spanish) starting in middle or high school. At a mean of 15.2 years of age, all multilinguals immigrated to the United States, where they had started learning and using English an average of 50% of the time (13 out of 15 participants went to high school or college in the U.S.; while all participants live and work in Romanian/English environments). Participants were recruited mainly through email, word of mouth, and at local church events. The lead author is a member of the community. Additionally, we selected only participants who rated themselves as highly proficient in all three languages but have Romanian as a dominant language. All multilingual participants reported speaking four or five languages; however, they also reported higher proficiency in three of these, commonly spoken by all multilinguals in the group, namely, Romanian (native language), Russian (an early learned L2), and English (a later learned second language that we will refer to as L3). The monolinguals were recruited to match the multilingual group as closely as possible on gender, age, and years of education, and were drawn from the Univeristy of Davis subject pool and included both undergraduate and graduate students. All monolingual participants indicated they have only used and have been able to communicate in English despite learning a foreign language in school for six months on average.

### Behavioral Data Acquisition

All participants completed a self-rating questionnaire. Along with background questions including the socioeconomic status of both the participants’ parents and the participants themselves, biological sex, and education level, the questionnaire used here asked participants to provide the age of acquisition, usage (including the environment in which they use a certain language, the amount of time spent speaking each language daily, etc.), and self-rated writing, reading, and speaking proficiency of each of the languages they know. (See Supporting Information, available at https://doi.org/10.1162/nol_a_00144 for additional information and/or additional background questionnaire data.)

All multilingual participants reported speaking four or five languages; however, they reported commonly speaking three of these, namely, Romanian (L1), Russian (L2), and English (L3). Hence, we assessed proficiency in these three languages within the multilingual groups, and in English for the monolinguals.

For the assessment of production proficiency and vocabulary knowledge, the participants were asked to complete the Multilingual Naming Test (MiNT; Ivanova et al., 2013). The original test consisted of 68 black-and-white line drawing images of different sources, an adaptation of a 32-image set was used for this study. Multilingual participants were asked to name the images in Romanian, Russian, and English, respectively.

Additionally, the vocabulary section of the Self-Administering Scale for Measuring Intellectual Impairment and Deterioration Test (Shipley, 1940), was used to assess the semantic and vocabulary knowledge of our participants. The English version of the test was translated into Romanian and Russian. The vocabulary items were matched as closely as possible in meaning, difficulty, and normed frequencies. Normed frequencies were computed for each translated item in Romanian and Russian from two large corpora. Romanian item frequencies were extracted from the Balanced Corpus of Romanian Language, containing approximately 5,500,000 tokens (Ciochina et al., 2020), and in Russian, from Wikipedia text extracted from the Wiki dump, approximately 442,400,562 tokens. Between Romanian and English, our final version of the tests had seven cognates out of a total of 40 words (∼15%); between the Russian and the English versions we had two cognates (∼5%). To mitigate these overlaps in languages, the proficiency tests were conducted on three different occasions (one for each language) for the multilinguals and on one single visit for the monolinguals. The language order was randomized across the multilingual participants.

Monolinguals completed the English version of the original test, while the multilinguals completed all three versions (i.e., the original English, the Romanian version, and the Russian version) on separate occasions. Lastly, a Grammatical Assessment Test (GAT) was constructed following a similar methodology to Linebarger et al. (1983), originally developed to assess comprehension failures in agrammatic aphasics. A new set of sentences for each of the languages (including the English version) was created, specifically designed to assess grammatical judgment and sentence comprehension of Romanian, Russian, and English (namely, 72 sentences for each language). Half of the sentence sets (36) were syntactically well formed, and 36 were systematically ill formed—representing 12 types of grammatical rule violations. Seven of the types were violations common to all three language structures (Romanian, Russian, and English), whereas the other five violation types were language-specific. All participants completed the English version of the GAT, while the multilinguals completed all three language versions on three different occasions (see Supporting Information for more information about the development/adaptation of each test to the three different languages). The language tests can be found at https://github.com/lmidriganciochina/Language-Proficiency-Tests.git.

### Image Acquisition

Participants were scanned with a Siemens 64-channel 3-Tesla Skyra MRI System (Siemens Healthcare, Erlangen, Germany) at the University of California, Davis. For the structural data, a T1-weighted imaging sequence using an MPRAGE (TfL) sequence with a voxel size = 0.9 × 0.9 × 0.9 mm^3^, FOV = 243 × 243 mm, 208 sagittal slices, 7° flip angle, TR = 2,500 ms, TI = 1,100 ms, and the TE = 4.44 ms. Bandwith 160 Hz/Px; GRAPPA = 2, 32 reference lines was used. A diffusion single-shot echo-planar imaging scan with 64 directions, b = 1,000 s/mm^2^, 106 × 106 matrix, FOV = 212 × 182 mm, isotropic 2 mm voxel size was acquired. A multiband acceleration factor of 2 allowed the acquisition of 64 slices in a time TR = 4,520 ms. The echo time was kept to a minimum of 84 ms by using a partial Fourier factor of 6/8. A single acquisition with the opposed phase encoding direction was used to correct the geometrical distortion induced by the inhomogeneous B0 field.

### Data Processing

The structural data preprocessing, image reconstruction, and volumetric and cortical parcellation were carried out with the *recon-all* command from the Freesurfer image analysis suite (Version 7.2.0), which is freely available software that can be found online and downloaded from https://surfer.nmr.mgh.harvard.edu/.

Diffusion-weighted images (DWIs) were preprocessed with the MRtrix3 software package, using the preprocessing pipeline recommended for FBA analysis of single-shell DWIs (Raffelt et al., 2017). The preprocessing steps included denoising (Veraart et al., 2016), eddy-current correction using FSL’s eddy (Andersson & Sotiropoulos, 2016; Smith et al., 2004), and bias field correction (Tustison et al., 2019). Global intensity normalization was performed using a group-wise (within the study) registration. To increase the anatomical contrast and improve spatial normalization and statistics, the images were upsampled to a voxel size of 1.25 mm^3^. A whole-brain mask was computed from the upsampled images. In order to enable an analysis of differences in the apparent fiber density (AFD) across subjects, a representative of the study population group average response was generated using the *tournier* function (Tournier et al., 2004), which enables the creation of a single-fiber WM function. Fiber orientation was estimated using the fiber orientation distribution (FOD) images (Raffelt et al., 2011) of all participants in the study. The group average response was computed by estimating the response function per subject with the multishell, multitissue CSD algorithm (*msmt_csd*) recommended even for single-shell data (Dhollander et al., 2016). These were then averaged across subjects. The location and orientation of the WM fixels were computed through the segmentation of each FOD lobe in the template (method described in Smith et al., 2013). All 30 participants’ FOD images were registered to the study FOD template using FOD-guided nonlinear registration (Raffelt et al., 2011; Raffelt et al., 2012) and then segmented to produce a set of discrete fixels (Raffelt et al., 2017; Smith et al., 2013). The resulting template is an unbiased group average and the estimated fixels are representative of the subjects in the study. FC, FD, and FDC were computed for each subject. Log (FC) was used to ensure that data are centered at about zero and are normally distributed. Finally, whole-brain fiber tractography was performed on the FOD template on 20 million streamlines that were further sifted to two million streamlines, using spherical deconvolution informed filtering (SIFT; Smith et al., 2015), to reduce bias in tractogram densities. All preprocessing steps were conducted using commands implemented in MRtrix3 (www.mrtrix.org) or using MRtrix3 scripts that interfaced with external software packages (Tournier et al., 2019).

### Statistical Analysis

#### Behavioral data analysis

Welch’s two-sample *t* tests were performed to assess differences in native language proficiency across groups. Paired sample t tests were performed to assess differences in proficiency across languages within the multilingual group. The test averages and statistical analysis were performed in R (R Core Team, 2020). Other summaries and descriptive analyses were performed in Excel.

#### MRI data analysis

Fixel-based statistical analysis and inferences were performed across groups for each WM fixel by a generalized linear model (GLM), comparing monolingual to multilingual participants. Mean-centered intracranial volume (ICV), computed with FreeSurfer image analysis suite (https://surfer.nmr.mgh.harvard.edu/), was included as a nuisance covariate in the GLM. The connectivity-based smoothing and statistical inference were done using connectivity-based fixel enhancement (CFE; Raffelt et al., 2015) on the template tractogram (i.e., 2 million streamlines). The default smoothing parameters described in Raffelt et al. (2015) were used (smoothing = 10 mm full-width and half maximum, C = 0.5, E = 2, and H = 3). Family-wise error (FEW) corrected *p* values were computed using non-parametric permutation testing of 5,000 permutations (Nichols & Holmes, 2002). Significant fixels (*p* < 0.05, FWE corrected) were displayed using the *mrview* tool in MRtrix3. Streamlines corresponding to significant fixels were additionally displayed on the template tractograms and were color-coded by effect size in terms of percentage relative to the control group (monolinguals) and by direction (left–right: red, inferior–superior: blue, anterior–posterior: green, at FWE-corrected *p* value of *p* < 0.05. All whole-brain fixel-based statistical analyses and visualizations were performed in MRtrix3.

For further analysis (e.g., correlations) the tracts that showed significant differences between the groups were categorized into WM tracts, using DWI atlases to guide categorization (Oishi et al., 2009; Wakana et al., 2004), and tracks were generated using the automated TractSeg method on the population’s FOD template. This method has been shown to provide reliable and accurate tract segmentation (Wasserthal et al., 2018). Tract segmentation and orientation maps were created for bundles that showed robust changes across groups in FD and FC, and have been associated with second language proficiency (namely, the bilateral IFOF, ILF, CC, and UF; e.g., Del Maschio et al., 2020; Elmer et al., 2011; Pliatsikas et al., 2015). For the correlation analysis, we restrict our analysis to changes in FD and FC and not the combined metric. The resulting pictograms were converted to fixel maps by cropping the template fixel mask to the fixels that were traversed by the streamlines that were part of the tract of interest, using the *tck2fixel* command in MRtrix (Tournier et al., 2019). The *mrstats* command in MRtrix (Tournier et al., 2019) was used to compute mean FD, FC (log), and FDC on each participant’s data, by providing the *-mask* option to the command. The output of the command gives a range of statistics (including the mean) that can be further used for statistical analysis. We acknowledge that our sample has limited power that may interfere with detecting the effects of interest (Munson & Hernandez, 2019). Hence, we have matched our participants on background variables as closely as possible and followed the suggested data processing and analysis pipelines.

## RESULTS

### Behavioral Data Analysis

Monolingual participants rated their English proficiency at 100%; multilingual participants rated their L1 proficiency at 100%, L2 at 72% (*SD* = 0.94), and L3 at 78% (*SD* = 0.91). Table 2 presents the summary of the background and self-rating assessments. According to the self-assessment, multilingual participants have had several years of L2 language immersion, with a mean of 11.3 years of daily use, and a mean of 12.5 years for the L3. L2 was used from a mean of 5.5 years to a mean of approximately 15 years, for a total of 9.5 years daily. Importantly, L2 was used daily up to the age of 15 (group average); thereafter, for a mean of 12.5 years, the participants started using only L3 daily as a second language, while L2 on occasion (i.e., some participants were still using the L2 daily). Hence, it is important to note, that although L2 and L3 have a similar amount of time of immersion and similar levels of proficiency, the two second languages were used in highly immersive contexts at different times in life. Additionally, all participants used the other known languages (see Table 2) on occasion, in different settings; assessing the proficiency for these languages was beyond the scope of this study.

**Table 2.** Participants’ background summary.

Monolingual participants						Multilingual participants														
*P*	Age	Bio sex	Years of ed.	English (native)		*P*	Age	Bio sex	Years of ed.	Romanian (native)		Russian			English			Other languages		
				AoA	SPR					AoA	SRP	AoA	SRP	Imm	AoA	SRP	Imm	Lang	AoA	SRP
1	28	F	21	birth	7	16	33	M	20	birth	7	5	6	28	14	6	23	It, Ukr	10	3, 4
2	23	M	17	birth	7	17	28	M	14	birth	7	6	4	23	17	6	13	Ukr, Fr	6, 13	5, 3
3	26	M	20	birth	7	18	27	F	14	birth	7	5	6	23	10	6	6	Ukr	4	3
4	23	F	18	birth	7	19	26	F	15	birth	7	4	4	15	21	4	12	Ukr, Fr	6, 10	3, 3
5	23	F	17	birth	7	20	31	M	15	birth	7	5	5	30	15	5	8	Ukr	10	5
6	25	M	19	birth	7	21	24	F	18	birth	7	2	5	11	13	6	11	Span	16	4
7	26	M	18	birth	7	22	27	F	17	birth	7	4	6	21	17	4	15	Span, Ger	9, 12	5, 2
8	26	M	18	birth	7	23	29	M	13	birth	7	6	6	28	17	5	23	Blr	0	5
9	23	F	17	birth	7	24	19	M	13	birth	7	5	5	5	20	4	14	Span	15	3
10	24	F	17	birth	7	25	30	M	15	birth	7	11	4	13	14	6	24	Ukr, Fr	7	4, 1
11	25	M	17	birth	7	26	26	M	15	birth	7	0	5	6	23	4	21	Ukr, Fr	11	4, 3
12	27	F	16	birth	7	=	27	M	15	birth	7	13	6	23	13	6	10	Ukr	9	4
13	22	M	16	birth	7	28	29	F	13	birth	7	12	4	27	6	6	16	Ukr	0	6
14	24	F	18	birth	7	29	29	F	20	birth	7	1	6	20	6	7	20	It, Fr	9, 13	3, 1
15	26	F	18	birth	7	30	29	M	20	birth	7	9	3	23	22	6	23	Fr, It	12	3, 3

*Note*. *P* = participant number; Years of Ed = years of education; Bio. sex = biological sex; Engl = English; Rom = Romanian; Russ = Russian; AoA = age of acquisition; SRP = Self-rated proficiency; Mono = monolingual; Multi = multilingual; Fr = French; Span = Spanish; Ger = German; Blr = Belarus; It = Italian.

The overall percentage errors for the language tests within the multilingual group was 10.02% (*SD* = 5.3%) for L1, 19.39% (*SD* = 8.5%) for L2, and 18.04% (*SD* = 9.6%) for L3, respectively. For the monolingual group L1, these were at 11.84% (*SD* = 5.3%; see Table 1 in the Supporting Information. A two-sample *t* test was carried out to compare proficiency between the groups for the native language. The difference between the L1 monolinguals and L1 multilinguals was not significant (t(28) = 1.05, *p* < 0.3; 95% CI, 2.56 to 0.83). Paired sample *t* tests were computed to further investigate differences between proficiency across languages within the multilingual group between the languages. Comparisons between the L1 and L2 scores (t(14) = 4.4; *p* < 0.001; 95% CI, 2.3 to 6.63) and L1 and L3 naming (t(14) = 4.2; *p* < 0.001; 95% CI, 1.87 to 5.76) were significant. There was not a statistical difference in scores between L2 and L3 (t(14) = 0.5, *p* = 0.6; 95% CI, 3.3 to 2.01). (See Table 3 for average and individual test results.)

**Table 3.** Proficiency data statistics.

	Proficiency data statistics											
	MINT			SHIPLEY			GAT			Avg proficiency		
	*p value*	*t stat*	*CI (95%)*	*p value*	*t stat*	*CI (95%)*	*p value*	*t stat*	*CI (95%)*	*p value*	*t stat*	*CI (95%)*
*L1(mono) > L1(multi)*	< 0.4	−1	−1.85 to 0.65	< 0.6	−1.9	−3.26 to 0.06	< 0.4	0.9	5.7 to 2.24	< 0.3	−1.05	−2.56 to 0.83
*L1(multi) > L2(multi)*	< 0.001*	7.2	4.45 to 8.21	< 0.2	1.3	−0.95 to 4.15	< 0.01*	3.2	1.78 to 9.14	< 0.001*	4.4	2.3 to 6.63
*L1(multi) > L3(multi)*	< 0.001*	4.04	2.87 to 9.38	< 0.01*	3.6	1.85 to 7.48	< 0.6	0.5	−2.4 to 3.7	< 0.001*	4.2	1.87 to 5.76
*L2(multi) > L3(multi)*	< 0.9	−0.1	−3.3 to 2.9	< 0.1	1.7	−0.72 to 6.85	< 0.1	0.5	2.44 to 3.78	< 0.6	−0.5	3.3 to 2.01

*Note*. *P* values were FDR corrected for the number of tests. Mono = monolingual; Multi = multilingual; GAT = Grammar test; MINT = Multilingual Naming Tests; Shipley = Self-Administering Scale for Measuring Intellectual Impairment and Deterioration; Lang. Use = language use.

### Behavioral Data Background Characteristics

A two-sample *t* test to asses a mean difference of age for monolinguals compared to multilinguals was significant (t(22) = −3; *p* < 0.01; 95% CI, −4.84 to −0.89). We, therefore, looked at correlations between the extracted WM tracts and age and found no significant associations.

Similarly, a two-sample *t* test revealed significant differences across groups for the years of education (t(22) 3, *p* < 0.01, 95% CI, 0.6 to −3.76); however, we found no significant correlation between years of education and any of the extracted fiber tracts. There was no difference across the groups on parental socioeconomic status (t(22) = −0.03, *p* < 0.7; 95% CI, −0.89 to 0.65) or participant socioeconomic status (t(22) = −2.08, *p* < 0.06; 95% CI, −1.45 to 0.01). We also found no association between socioeconomic status and the WM tracts. See Table 4 for the correlation results between selected WM tracts and participant background characteristics.

**Table 4.** White matter correlation analyses with background characteristics.

	Monolinguals background variables								Multilinguals background variables							
	SESp		SESs		EDU		Age		SESp		SESs		EDU		Age	
	R	*p*	R	*p*	R	*p*	R	*p*	R	*p*	R	*p*	R	*p*	R	*p*
CC (FD)	0.03	0.9	−0.3	0.2	0.07	0.8	−0.2	0.5	−0.3	0.2	−0.2	0.5	−0.13	0.65	−0.3	0.1
CC (FC)	0.04	0.8	−0.07	0.8	−0.03	0.23	0.25	0.4	−0.1	0.6	−0.03	0.9	−0.05	0.8	−0.07	0.8
l0. IFOF (FD)	0.03	0.9	−0.3	0.3	0.07	0.8	−0.01	0.9	0.26	0.35	0.2	0.5	0.34	0.2	0.04	0.88
l0. IFOF (FC)	−0.2	0.5	−0.03	0.3	−0.3	0.1	−0.03	0.8	0.14	0.6	−0.03	0.92	0.3	0.25	−0.12	0.66
r0. IFOF (FD)	−0.2	0.6	−0.2	0.5	−0.4	0.1	−0.2	0.5	0.42	0.1	0.26	0.34	0.4	0.1	0.24	0.38
r0. IFOF (FC)	−0.35	0.2	−0.32	0.2	−0.3	0.2	0.2	0.5	0.2	0.5	0.05	0.85	0.4	0.1	0.01	0.96
l0. ILF (FD)	0.21	0.4	−0.19	0.5	0.29	0.29	−0.01	0.9	0.12	0.7	−0.03	0.9	0.21	0.45	−0.14	0.6
l0. ILF (FC)	0.01	0.9	−0.14	0.6	−0.32	0.25	0.04	0.87	0.3	0.3	−0.03	0.9	0.31	0.26	−0.07	0.8
r0. ILF (FD)	0.21	0.45	−0.02	0.55	−0.2	0.5	−0.4	0.1	0.45	0.08	0.21	0.4	0.31	0.26	0.31	0.3
r0. ILF (FC)	−0.13	0.6	−0.28	0.31	−0.2	0.4	0.25	0.36	0.4	0.14	0.11	0.7	0.44	0.09	0.09	0.7
l0. UF (FD)	−0.3	0.4	−0.06	0.8	0.3	0.2	0.3	0.1	0.18	0.5	−0.07	0.8	0.3	0.3	0.1	0.6
r0. UF (FC)	−0.5	0.05*	−0.2	0.4	−0.36	0.2	−0.03	0.9	0.28	0.3	0.04	0.8	0.4	0.08	0.05	0.85

*Note*. FD = fiber density; FC = fiber cross-section; l = left; r = right; CC = corpus callosum; IFOF = inferior fronto-occipital fasciculus; ILF = inferior longitudinal fasciculus; UF = uncinate fasciculus. R = correlation coefficient; *p* = *p* value of the correlation test. SESp = parental socieoeconomical status; SESs = participant socioeconomical status; EDU = education; Age = age of participant at scan.

### Whole-Brain Analysis Results

#### White matter differences for a comparison of multilinguals > monolinguals

**Figure 1.**
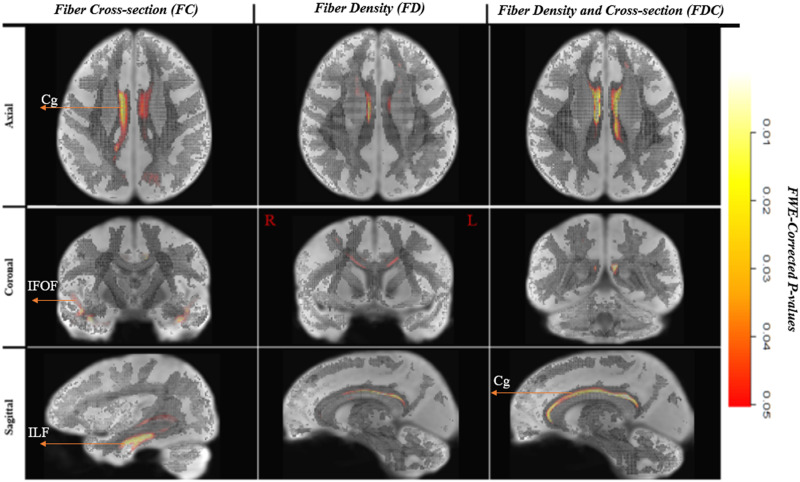
White matter significant fixels: multilinguals > monolinguals. Fixels with a significant (*p* < 0.05) higher fiber-bundle cross-section, fiber density, and fiber density and cross-section for multilinguals compared to monolinguals. Fixels are color-coded by family-wise error (FWE) corrected *p* values and overlaid on the white matter study template map. IFOF = intrerior fronto-occipital fasciculus, ILF = inferior longitudinal fasciculus, Cg = Cingulum.

Figure 1 shows fixels that had significantly higher FC, FD, and FDC for the multilinguals compared to the monolinguals. The microstructural differences in FD were limited to a segment of the right anterior cingulum bundle. The differences were up to > 15% higher for the multilingual group.

On the other hand, macrostructural differences in FC were more pronounced (up to > 40%) and included the bilateral cingulum (Cg), the anterior sections of the ventral language pathway, including bilateral IFOF, the bilateral ILF, and right UF. The segments that showed significant differences were the anterior sections, passing through the anterior temporal lobe. Fiber bundles showed more robust differences in the right hemisphere. Additionally, one small segment of the dorsal pathway, namely, the temporo-parietal portion of the right superior longitudinal fascicle (SLFtp), overlapping with the posterior segment of the AF showed higher FC values for the multilinguals compared to the monolinguals. Lastly, the right inferior cerebellar peduncle (ICP) showed a higher fiber cross-section in the multilinguals.

**Figure 2.**
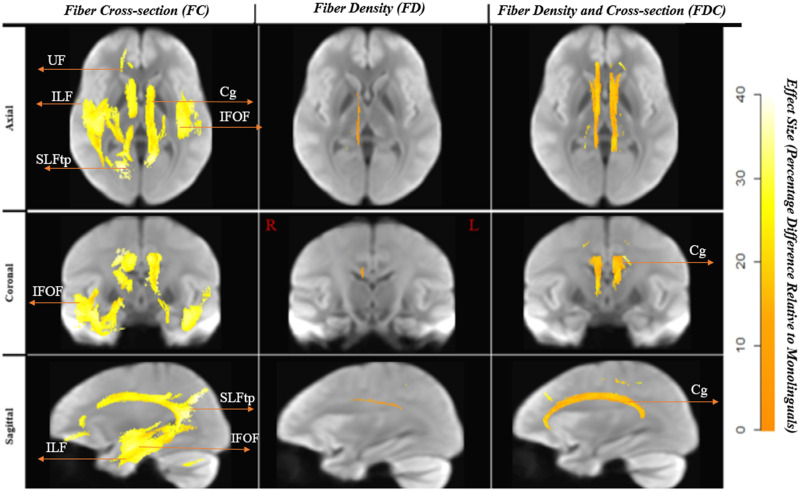
Tractograms of significant white matter fixels: multilinguals > monolinguals. Effect sizes are expressed as a percentage increase relative to the monolingual group. To enable the visualization of all the significant fixels in 3D, streamlines from the template-derived whole-brain tractogram were cropped to include streamline points that correspond to significant fixels (FWE-corrected *p* value < 0.05). For a direct comparison of effect sizes across FD, FC, and FDC, streamlines shown correspond to significant fixels from all three analyses combined (i.e., the union of FD, FC, FDC). IFOF = intrerior fronto-occipital fasciculus, ILF = inferior longitudinal fasciculus, SLFtp = superior longitudinal fasciculus (temporal segment), UF = uncinate fasciculus, Cg = Cingulum.

**Figure 3.**
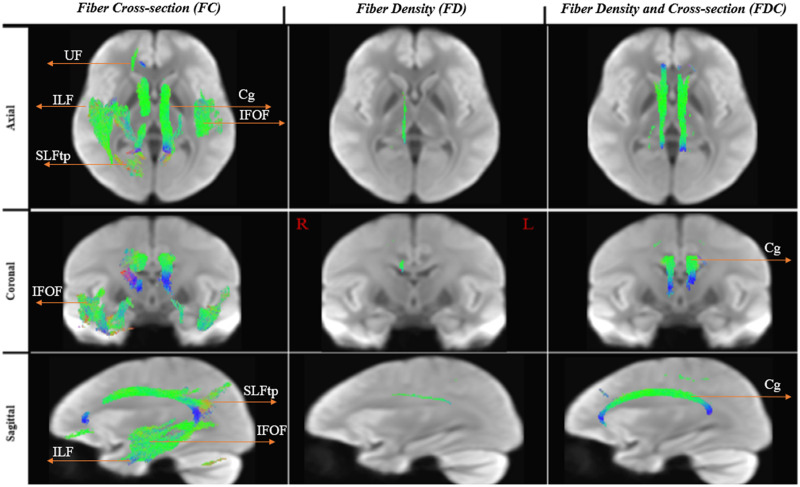
Reconstructed white matter pathways of significant fixels: multi > mono. White matter pathways that showed significantly higher FD, FC, and FDC in the multilinguals compared to the monolinguals. To enable the visualization of all significant fixels in 3D (i.e., not just a 2D slice), streamlines from the template-derived whole-brain tractogram were cropped to include streamline points that correspond to significant fixels (FWE-corrected *p* value < 0.05), and colored by direction (red: left–right plane, indicating fibers going horizontally from the left toward the ride side of the brain; blue: inferior–superior, indicating fibers going vertically along the inferior toward the superior side of the brain; green: anterior–posterior plane, indicating fibers going horizontally from the back toward the front of the brain). IFOF = intrerior fronto-occipital fasciculus, ILF = inferior longitudinal fasciculus, SLFtp = superior longitudinal fasciculus (temporal segment), UF = uncinate fasciculus, Cg = Cingulum.

The FDC measure was significantly higher (effect size of > 30%) for the multilinguals in the Cg. The largest differences for this comparison were noted in the FC (see Figure 2). Figure 3 displays the streamlines corresponding to the significant fixels, derived from the whole brain tractogram, color-coded by direction (red: left–right, blue: inferior–superior, green: anterior–posterior).

#### White matter differences for a comparison of multilinguals < monolinguals

**Figure 4.**
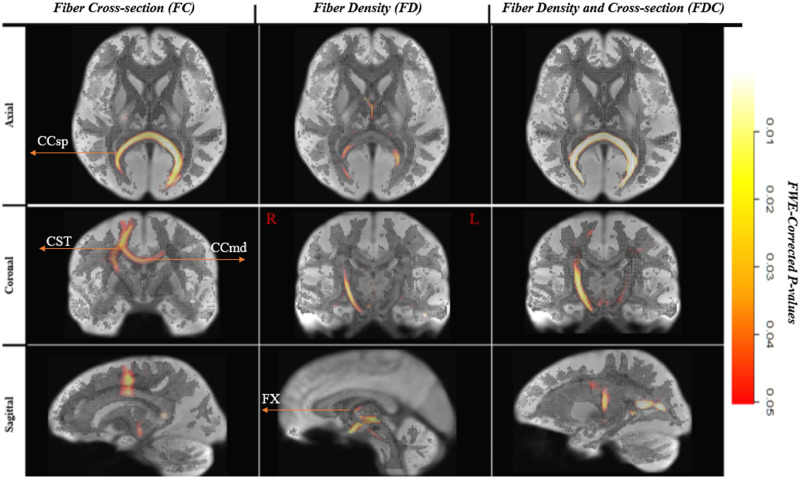
White matter significant fixels: monolinguals > multilinguals. Fixels with a significant (*p* < 0.05) lower fiber-bundle cross-section, fiber density, and fiber density and cross-section for multilinguals compared to monolinguals. Fixels are color-coded by family-wise error (FWE) corrected *p* values and overlaid on the white matter template map. CCsp = corpus callosum (splenium), CCmd = corpus callosum (middle segment), CST = cortico-spinal tract, FX = Fornix.

**Figure 5.**
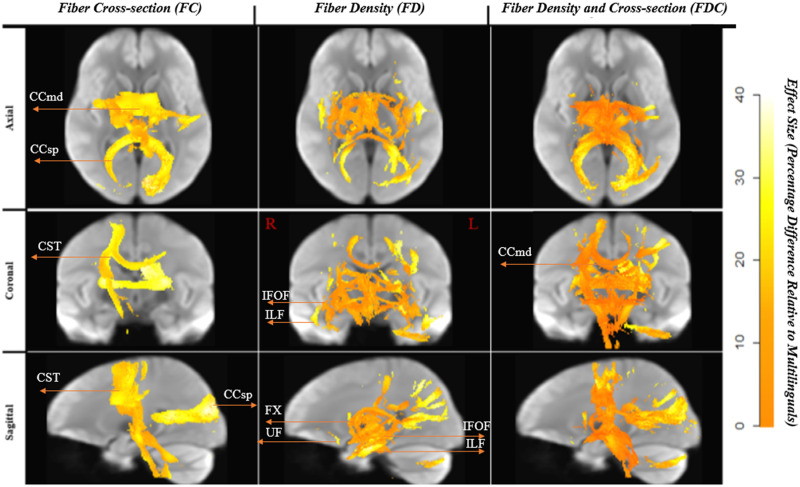
Tractograms of significant white matter fixels: monolinguals > multilinguals. Effect sizes expressed as a percentage decrease relative to the control group. To enable the visualization of all the significant fixels in 3D, streamlines from the template-derived whole-brain tractogram were cropped to include streamline points that correspond to significant fixels (FWE-corrected *p* value < 0.05). For a direct comparison of effect sizes across FD, FC, and FDC, streamlines shown correspond to significant fixels from all three analyses combined (i.e., the union of FD, FC, FDC). IFOF = intrerior fronto-occipital fasciculus, ILF = inferior longitudinal fasciculus, SLFtp = superior longitudinal fasciculus (temporal segment), UF = uncinate fasciculus, CCsp = corpus callosum (splenium), CCmd = corpus callosum (middle segment), CST = cortico-spinal tract, FX = Fornix.

**Figure 6.**
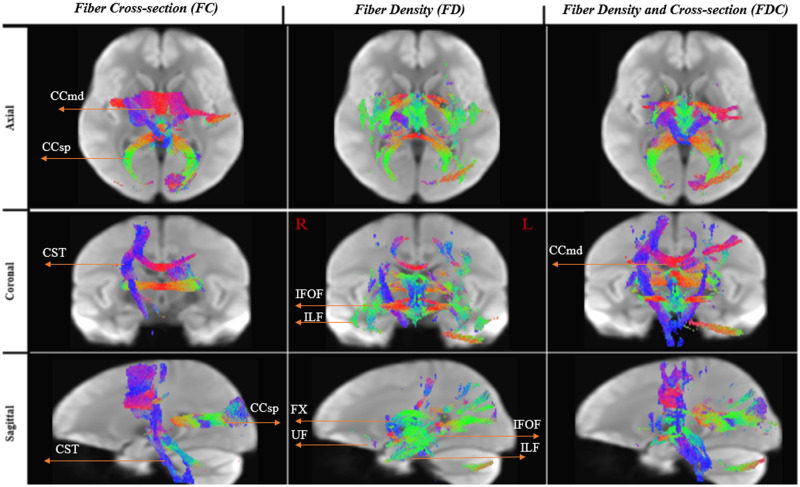
Reconstructed white matter pathways of significant fixels: mono > multi. White matter pathways that showed significantly higher FD, FC, and FDC in the monolinguals compared to multilinguals. To enable the visualization of all significant fixels in 3D (i.e., not just a 2D slice), streamlines from the template-derived whole-brain tractogram were cropped to include streamline points that correspond to significant fixels (FWE-corrected *p* value < 0.05), and colored by direction (red: left–right plane, indicating fibers going horizontally from the left toward the ride side of the brain, blue: inferior–superior, indicating fibers going vertically along the inferior toward the superior side of the brain, green: anterior–posterior plane, indicating fibers going horizontally from the back toward the front of the brain). For a direct comparison of effect sizes across FD, FC, and FDC, streamlines shown correspond to significant fixels from all three analyses combined (i.e., the union of FD, FC, FDC). IFOF = intrerior fronto-occipital fasciculus, ILF = inferior longitudinal fasciculus, UF = uncinate fasciculus, CCsp = corpus callosum (splenium), CCmd = corpus callosum (middle segment), CST = cortico-spinal tract, FX = Fornix.

Figure 4 shows plots of fixels that had significant (FEW, *p* < 0.05) lower FC, FD, and FDC for the multilingual group compared to the monolinguals. These are color-coded by FWE corrected *p* values and overlaid on the WM template map. Differences were observed across different fiber bundles, with some fibers (e.g., the CC [splenium] exhibiting lower FD up to < 46% (see Figure 5) in the multilinguals compared to monolinguals. Lower FD was observed in the medial pathway involved in language processing and control, namely, the CC radiation that passes through the splenium and the midbody, as well as anterior portions (passing through the temporal lobe) of bundles of the ventral pathway, namely, the bilateral IFOF, ILF, and left UF. FD was also lower in the left ICP, medial segments of the right corticospinal tract, and bilateral fornix (see Figure 5 and Figure 6).

Macrostructural differences in the FC were more pronounced and more spatially restricted to delimited fiber bundles, including the right cortico-spinal tract (CST), the radiation passing through the central body of the CC, and the segment passing through the CC splenium. Figure 5 presents the results using template-driven streamlines. The streamlines presented here correspond to all the WM fixels that exhibited significantly lower FD, FC, and FDC in the multilinguals. These are projected on top of the WM population template and are color-coded by percent effect size relative to the monolinguals. Figure 6 displays the same streamlines, color-coded by direction (i.e., red: left–right, blue: inferior–superior, green: anterior–posterior).

Combined macro- and microstructural (i.e., FDC) differences were observed, as expected, in fibers that showed differences in the FD and/or in FC, with the exception of the ventral pathway bundles that showed a difference in FD (i.e., anterior portions of the IFOF, ILF, and UF). These include the bilateral CST (with rightward asymmetry), the radiation passing through the central body of the CC and the CC isthmus, as well as the fornix, and the left ICP.

Noteworthy, the ventral pathway fiber bundles (i.e., IFOF, IFL, UF) showed a very specific change with higher FC (higher fiber bundle size perpendicular to the length of the fiber) and showed lower FD (lower volume of intra-axonal restricted water) values for the multilinguals. They did not show significant differences in the combined FDC measure. Table 5 lists a summary of the significant results.

**Table 5.** Fixel-based analysis results of white matter morphometry.

*Multilinguals > Monolinguals*	
*FD*	- right Cg
*FC*	- bilateral Cg
	*ventral pathway*
	- bilateral IFOF
	- bilateral ILF
	- right UF
	*dorsal pathway*
	- right SLFtp/AF
	- right ICP
*FDC*	- bilateral Cg

*Multilinguals < Monolinguals*	
*FC*	*medial pathway*
	- CC midbody
	- CC splenium
	*sensory-motor mapping*
	- right CST
	- bilateral FX
*FD*	*medial pathway*
	- CC midbody
	- CC splenium
	*sensory-motor mapping*
	- CST
	- bilateral FX
	- left ICP
	*ventral pathway*
	- bilateral IFOF
	- bilateral ILF
	- left UF
*FDC*	*medial pathway*
	- C midbody
	- CC splenium
	*sensory-motor mapping*
	- right CST
	- bilateral FX
	- left ICP

*Note*. Fiber bundles showing significant results in the fixel-based analysis for the multilinguals versus the monolingual group. Cg = cingulum, IFOF = inferior fronto-occipital fasciculus, ILF = inferior longitudinal fasciculus, UF = uncinate fasciculus, SLFtp/AF = superior longitudinal fasciculus, temporal segment/arcuate fasciculus, ICP = inferior cerebellar peduncle, CC = corpus callosum, FX = fornix, CST = corticospinal tract.

### Additional Statistical Testing

Lastly, we have looked at correlations between the extracted WM in the CC, IFOF, ILF, and UF (i.e., predefined ROIs that showed significant differences across groups), generally associated with brain changes in bilinguals (e.g., Del Maschio et al., 2020; Elmer et al., 2011; Pliatsikas et al., 2015) and proficiency tests data (see Table 6 and Table 7), as well as age of acquisition, and language use (see Table 8).

**Table 6.** White matter correlation analyses with language test data: L2 and L3.

	Russian, early L2								English, late L3							
	MINT		SHIPLEY		GAT		Avr. Prof		MINT		SHIPLEY		GAT		Avr. Prof	
	R	*p*	R	*p*	R	*p*	R	*p*	R	*p*	R	*p*	R	*p*	R	*p*
*CC (FD)*	−0.32	0.2	−0.5	0.07	0.18	0.5	−0.23	0.4	−0.3	0.3	0.3	0.3	0.02	0.9	−0.13	0.9
*CC (FC)*	0.32	0.2	−0.23	0.4	0.4	0.1	0.2	0.5	0.4	0.1	0.2	0.5	0.3	0.3	0.14	0.6
*l. IFOF (FD)*	−0.6	0.01*	−0.5	0.05*	−0.35	0.19	−0.6	0.02*	−0.5	0.07	0.16	0.57	0.05	0.8	−0.12	0.68
*l. IFOF (FC)*	0.3	0.4	−0.03	0.9	0.18	0.5	0.16	0.6	0.37	0.17	0.18	0.52	0.03	0.89	−0.3	0.3
*r. IFOF (FD)*	−0.4	0.14	−0.54	0.03*	−0.5	0.08	−0.6	0.02*	−0.06	0.8	−0.43	0.1	−0.05	0.86	0.006	0.98
*r. IFOF (FC)*	0.13	0.6	−0.1	0.73	0.13	0.6	−0.06	0.8	0.32	0.25	0.3	0.3	−0.02	0.9	−0.3	0.33
*l. ILF (FD)*	−0.2	0.52	−0.04	0.88	0.001	0.99	−0.08	0.77	0.01	0.98	−0.25	0.35	0.08	0.76	−0.02	0.9
*l. ILF (FC)*	0.35	0.2	0.16	0.56	0.28	0.31	0.32	0.23	0.6	0.05*	−0.17	0.53	0.14	0.62	0.3	0.3
*r. ILF (FD)*	−0.02	0.95	−0.17	0.55	0.02	0.95	−0.07	0.8	0.4	0.1	−0.27	0.33	0.17	0.55	0.14	0.6
*r. ILF (FC)*	0.09	0.7	−0.01	0.95	0.21	0.44	0.12	0.7	0.4	0.16	−0.41	0.14	0.07	0.78	0.01	0.95
*l. UF (FD)*	−0.4	0.2	−0.2	0.46	−0.6	0.02*	−0.5	0.06	−0.06	0.64	0.4	0.14	−0.07	0.8	−0.1	0.6
*r. UF (FC)*	0.16	0.6	−0.07	0.78	0.2	0.5	0.1	0.6	0.52	0.04*	−0.34	0.22	0.07	0.8	−0.1	0.6

*Note*. FD = fiber density; FC = fiber cross-section; l = left; r = right; CC = Corpus Callosum; IFOF = Inferior Fronto-Occipital Fasciculus; ILF = Inferior Longitudinal Fasciculus; UF = Uncinate Fasciculus. R = correlation coefficient; *p* = the *p* value of the correlation test; MINT = Multilingual Naming Test; SHIPLEY = Self-Administering Scale for Measuring Intellectual Impairment and Deterioration Test (vocabulary section); GAT = Grammatical Assessment Test; Lang Use = Language Use.

**Table 7.** White matter correlation analyses with language test data: L1.

	Monolinguals, native L1								*Multilinguals, native L1*							
	MINT		SHIPLEY		GAT		Avg. Prof		MINT		SHIPLEY		GAT		Avg. Prof	
	R	*p*	R	*p*	R	*p*	R	*p*	R	*p*	R	*p*	R	*p*	R	*p*
CC (FD)	0.34	0.2	0.5	0.05*	0.04	0.8	0.16	0.56	−0.1	0.7	−0.2	0.4	−0.02	0.9	−0.13	0.6
CC (FC)	−0.1	0.7	−0.5	0.1	0.43	0.1	−0.5	0.06	0.2	0.4	0.1	0.7	0.3	0.3	0.33	0.23
l. IFOF (FD)	−0.08	0.7	0.4	0.1	−0.13	0.6	0.005	0.9	−0.01	0.95	−0.2	0.4	−0.3	0.4	−0.3	0.3
l. IFOF (FC)	−0.04	0.87	−0.3	0.3	−0.23	0.4	−0.3	0.3	0.5	0.05*	0.2	0.6	0.3	0.3	0.4	0.1
r. IFOF (FD)	0.03	0.9	0.34	0.2	0.2	0.4	0.3	0.3	0.09	0.7	−0.08	0.7	−0.06	0.8	0.4	0.1
r. IFOF (FC)	0.01	0.9	−0.4	0.1	−0.2	0.5	−0.3	0.3	0.6	0.02*	0.07	0.7	0.24	0.4	−0.15	0.15
l. ILF (FD)	0.05	0.8	0.5	0.05*	−0.07	0.8	0.09	0.7	0.009	0.97	−0.09	0.7	−0.28	0.3	−0.3	0.3
l. ILF (FC)	−0.03	0.89	−0.3	0.2	−0.5	0.07	−0.48	0.06	0.4	0.15	0.4	0.15	0.28	0.3	0.6	0.05*
r. ILF (FD)	−0.07	0.8	0.6	0.02 *	0.13	0.6	0.26	0.34	−0.06	0.8	0.34	0.2	0.005	0.9	0.12	0.7
r. ILF (FC)	0.001	0.99	−0.04	0.1	−0.32	0.2	−0.4	0.1	0.52	0.05*	0.23	0.4	0.23	0.4	0.4	0.1
l. UF (FD)	−0.3	0.35	0.27	0.33	−0.17	0.5	−0.09	0.72	−0.02	0.9	0.001	0.99	−0.1	0.7	0.09	0.7
r. UF (FC)	−0.2	0.5	−0.43	0.1	−0.3	0.4	−0.04	0.2	0.55	0.03*	0.25	0.4	0.2	0.6	0.4	0.1

*Note*. FD = fiber density; FC = fiber cross-section; l = left; r = right; CC = corpus callosum; IFOF = inferior fronto-occipital fasciculus; ILF = inferior longitudinal fasciculus; UF = uncinate fasciculus. R = correlation coefficient; *p* = the *p* value of the correlation test; MINT = Multilingual Naming Test; SHIPLEY = Self-Administering Scale for Measuring Intellectual Impairment and Deterioration Test (vocabulary section); GAT = Grammatical Assessment Test; Avg. Prof = average proficiency.

**Table 8.** White matter correlation analyses with age of acquisition and language use.

	Native L1		Russian L2				English L3			
	Lang. use		AoA		Lang. use		AoA Engl		Lang. use	
	R	*p*	R	*p*	R	*p*	R	*p*	R	*p*
CC (FD)	−0.4	0.15	−0.3	0.27	−0.17	0.5	0.15	0.58	−0.02	0.9
CC (FC)	−0.07	0.8	−0.12	0.67	−0.2	0.4	0.28	0.31	0.12	0.6
l. IFOF (FD)	0.04	0.9	0.009	0.97	0.32	0.24	−0.13	0.65	−0.07	0.78
l. IFOF (FC)	−0.12	0.7	−0.03	0.93	−0.06	0.8	0.006	0.98	0.03	0.9
r. IFOF (FD)	0.24	0.4	−0.17	0.55	0.33	0.23	0.05	0.86	0.21	0.4
r. IFOF (FC)	0.01	0.96	−0.01	0.97	0.06	0.8	−0.02	0.95	0.04	0.86
l. ILF (FD)	−0.15	0.6	0.23	0.41	0.23	0.4	−0.23	0.4	−0.07	0.81
l. ILF (FC)	−0.07	0.8	−0.09	0.74	−0.09	0.7	−0.1	0.0.7	0.28	0.3
r. ILF (FD)	0.31	0.26	0.12	0.66	0.12	0.7	−0.8	0.0.7	0.57	0.02*
r. ILF (FC)	0.09	0.7	0.09	0.73	0.09	0.7	−0.1	0.7	0.3	0.2
l. UF (FD)	0.1	0.7	0.7	0.002*	0.005	0.9	−0.04	0.16	0.12	0.7
r. UF (FC)	0.05	0.8	0.02	0.9	0.07	0.8	−0.05	0.85	0.16	0.6

*Note*. FD = fiber density; FC = fiber cross-section; l = left; r = right; CC = corpus callosum; IFOF = inferior fronto-occipital fasciculus; ILF = inferior longitudinal fasciculus; UF = uncinate fasciculus; R = correlation coefficient; *p* = *p* value of the correlation test; AoA = age of acquisition; Lang use = language use.

## DISCUSSION

### Behavioral Data

The results of the proficiency tests used in this study showed similar proficiency for both groups in the native languages; both groups showed excellent performance in all three tests, as well as 100% self-rated proficiency. For the multilingual group, the overall performance of the proficiency tests indicated that participants showed high proficiency in all languages; however, statistically different results were obtained between native versus non-native languages. The multilingual participants had equal proficiency in the L1 with the monolinguals and slightly lower proficiency in L2 and L3. Importantly proficiency measures for L2 (Russian) and L3 (English) were equivalent in the multilingual group. Overall, our multilingual group demonstrated close to native-like proficiency within at least three languages and long-term immersion within multilingual contexts.

### Fixel-Based Analyses

In this study, we applied a recently developed method to investigate WM changes in multilingual speakers compared to monolinguals. The first hypothesis we investigated in this study was increased WM integrity in the anterior regions of the brain (Grundy et al., 2017), in pathways involved in language processing, namely the dorsal pathway fiber bundles (SLF, AF) and the ventral pathway fiber bundles (IFO, ILF, UF).

#### Ventral pathways

Higher FC (fiber bundle size perpendicular to the length of the fiber) and lower FD (a lower volume of intra-axonal restricted water) values were observed for the multilinguals in the anterior portions of the fiber bundles of the ventral language processing pathways (namely, the IFOF, ILF, UF). These results are partially supporting our hypothesis, as we expected higher values in both FC and FD.

The ventral pathway for language processing realizes the communication between regions supporting sound-to-meaning mapping, language comprehension, and basic syntactic processing (Brauer et al., 2013; Friederici & Gierhan, 2013; Hickok & Poeppel, 2007; Leclercq et al., 2010; Mandonnet et al., 2007; Martino et al., 2013; Pliatsikas, 2019; Tremblay & Dick, 2016). Differences observed here are suggestive of an increase in the efficiency of information transfer across such brain regions as a response to the demand of accommodating the sound categories, the concepts, and the size of the vocabulary of multiple languages. Although FBA metrics and metrics derived with the tensor models, cannot be meaningfully directly compared, changes in FA, and MD of the WM of the dorsal and ventral pathways for bilinguals compared to monolinguals have been reported in numerous studies (e.g., Luk et al., 2011; Pliatsikas et al., 2015; Rahmani et al., 2017).

We observe differences in the bilateral portions of the IFOF and ILF passing through the temporal lobe. According to recent dual-stream language processing models, the speech recognition process, as well as some of the combinatorial processes involved in mapping the speech input into semantic representations are bilaterally organized (Friederici & Gierhan, 2013; Hickok & Poeppel, 2004, 2007). The task of accommodating phonetic and semantic information in multiple languages (e.g., computing the representation of distinctive features of the speech signal in the appropriate language, its syllabic structure, prosodic characteristics, and the process of mapping this information to appropriate concepts), may lead to an increase in communication efficiency within temporal lobe regions for speakers of multiple languages.

#### Dorsal pathways

Consistent with our hypothesis, increases in FC, in a short segment of the SLFtp were observed for the multilinguals compared to the monolinguals.

The dorsal pathway is believed to be involved in top-down complex syntactic processing (i.e., anchored by the AF), and input-driven auditory-to-motor mapping, supported by the SLF (Friederici, 2012; Tremblay & Dick, 2016). It is worth mentioning that the SLFtp (Tremblay & Dick, 2016) has been identified to overlap with the AF (Catani & de Schotten, 2008; Makris et al., 2005). The portion of the dorsal stream connecting the superior temporal cortex to Broca’s area supports the processing of syntactically complex sentences, by predicting the incoming input by providing top-down feedback (Friederici & Gierhan, 2013). Changes in FA values within the SLF have been previously reported in bilinguals compared to monolinguals (Luk et al., 2011; Mamiya et al., 2016). For example, Pliatsikas et al. (2015) showed higher FA values in sequential bilinguals, highly immersed in a second language environment, and suggest that changes in WM integrity between regions involved in language processing are the result of continuous cognitive stimulation from actively using multiple languages. Multilinguals do not only have to compute the appropriate syntactic construction for each language’s sentences but also potentially use different strategies for computing this information (Kovelman et al., 2008). While in English the speaker may rely on word order to compute the meaning of the sentence, in Romanian and Russian, the morphological markers may provide more reliable information for computing the overall sentence meaning. These processes may lead to increased efficiency within WM bundles supporting different syntactic processing strategies depending on the language.

We observe a very specific type of structural change in our sample, namely, higher FC (number of voxels/pixels occupied by the bundle) and lower FD (volume of intra-axonal restricted water), while no changes in the total FDC were generally observed within language pathways. Since the local FD metric represents an indirect measure of the axonal matter along the fiber tract, within a voxel unit (Dhollander et al., 2021) it is heavily influenced by the extracellular space within the unit. Additionally, the FD metric cannot distinguish between the number of axons or the axonal diameter. These patterns of results can be due to several biological changes that influence the FBA metrics (for a discussion on how to interpret the FD, FC, and FDC values, see Dhollander et al., 2021). However, an appropriate interpretation for this specific population corresponds to two different scenarios: (1) The volume fraction of the extra-axonal space has changed (e.g., causing the FC volume to expand, consistent with increased FC) without changing the intra-axonal volume of restricted water (i.e., number of axons, or axonal diameter, as measured by the FD metric), further resulting in an unobservable change in the FDC. Whether these changes are associated with processes of mylenogenesis cannot be determined with this metric, since it is not sensitive to myelin (Dhollander et al., 2021); however, along with various other microstructural changes, the myelin may affect the structure of the extracellular space (Mancini et al., 2020). (2) A change in the number of axons, accompanied by myelination, results in a total lower FD, while the volume occupied by the FC has increased and the FDC remains similar across groups (Dhollander et al., 2021). Although the exact biological changes associated with these patterns of results are not possible to determine given the limitation of the DWI techniques, it is important to note that newer models, such as FBA may help reconcile some of the mixed patterns of results that we observe in the bilingual literature (i.e., increases and decreases in the tensor-based metrics). There are specific changes that we observe within the same fiber pathways, namely, higher FC (number of voxels/pixels occupied by the bundle) and lower FD (volume of intra-axonal restricted water). Such patterns of changes may be undetectable with the traditional DTI metrics, since the specific morphological WM changes associated with the metrics of FA, MD, etc., are not distinguishable.

The observed increased WM microstructure in the frontal regions of the brain is in agreement with the predictions of the DRM, and the BAPSS, which hold that with increased proficiency bilinguals become more efficient by facilitating communication between anterior and subcortical/posterior regions through white matter diffusion increases. With high proficiency, a shift from devoting resources to the anterior regions should be noted toward more subcortical and posterior brain regions, with enhanced white matter integrity in the anterior parts of the brain (Grundy et al., 2017).

#### Medial pathways

A second hypothesis investigated in this study was reduced FD, FC, and FDC values in the CC (Pliatsikas, 2020). Two different segments of the corpus callosum showed decreases in all three morphometry measures FD, FC, and FDC, for multilinguals compared to monolinguals. The splenium of the CC contains fibers connecting the parietal temporal language-related regions (Coggins et al., 2004). The CC has been reported to be involved in the interhemispheric transfer of auditory information (Pollmann et al., 2002) and the development of verbal abilities (Nosarti et al., 2004). A previous electroencephalography (EEG) study discusses the functional relevance of the posterior CC in the processing of suprasegmental prosodic information and syntactic information in patients with posterior callosal lesions. Compared to the controls, which displayed an N400 effect for prosodically mismatched verb-argument structures, signaling lexical integration difficulty for unexpected argument structure for certain verb classes (e.g., a structure in which a direct object follows an intransitive verb) the patients did not exhibit a prosody-induced N400 effect (Friederici et al., 2007). The authors underline the relevance of the posterior part of the CC (including the isthmus) in the interplay between the verb argument and the relevant syntactic information provided by the prosody. Multilinguals, compared to monolinguals, have increased demand in computing the syntactic structure by using different prosodic information (i.e., appropriate for the language spoken in a specific interactional context) for adequate interpretation of the sentences’ meanings.

While reports of increases in WM integrity associated with increased communication efficiency between brain regions are well attested (Dhollander et al., 2021; García-Pentón et al., 2014; Mohades et al., 2015; Pliatsikas et al., 2015; Qi et al., 2015; Schlegel et al., 2012), opposite patterns have also been reported (Pliatsikas, 2020), especially in participants with a higher proficiency level in a second language (Singh et al., 2018), and/or with a long-time history of second language use (i.e., sequential bilinguals, in Mohades et al., 2012; lifelong bilinguals in Gold et al., 2013).

Further insights supporting lower brain morphology values have been attested in groups of simultaneous interpreters, which often are in disagreement with trends reported in bilingual literature. In line with studies of highly proficient bilinguals that have a long history of second language use, interpreters have shown reduced values of anisotropy in several WM tracts, including the CST, and genu, body, and splenium of the CC (Elmer et al., 2011). The authors attribute the observed reductions in FA to adaptations due to specific demands of interpreting. However, one study (Midrigan-Ciochina et al., 2023) showed reductions in gray matter in a multilingual group compared to monolinguals, similar to previously reported changes in simultaneous interpreters (Elmer et al., 2011).

Unrelated to the hypothesis investigated in this study, a fiber bundle that showed consistently higher morphometry values (for FD, FC, and FDC) in multilinguals compared to monolinguals was the bilateral Cg. The Cg bundle is a WM tract that connects the frontal, temporal, and parietal regions, as well as the cingulate gyrus to the subcortical regions (Bubb et al., 2018). Accumulative evidence from both nonhuman and healthy and clinical human populations has implicated the Cg in tasks involving working memory, attention, and executive functions. (For a detailed review, see Bubb et al., 2018.) The Cg has been documented in only very few studies, to show WM differences in bilinguals. Rahmani et al. (2017) looked at a group of bilinguals compared to monolinguals and found an increase in quantitative anisotropy in the bilingual group. Another study reported an association between the FA in the dorsal and anterior segments of the Cg with the level of L2 usage of a group of native Italian adults—speakers of English as a second language (Del Maschio et al., 2020). There is ample evidence showing the involvement of the anterior cingulate cortex (ACC) in linguistic and nonlinguistic activities that require conflict and error monitoring is unequivocal (Abutalebi & Green, 2016; Seo et al., 2018). Similarly, the involvement of the subcortical areas (linked to the ACC through the Cg) in language control processes is unequivocal (e.g., Abutalebi & Green, 2016; Pliatsikas, 2019). The increased demands in control in multilinguals related to juggling multiple languages and resolving interference from inappropriate languages, impose increases in recruitment of areas related to control in bi/multilinguals and may lead to restructuring in pathways related to language control, such as the Cg bundle.

Lastly, our results show overall lower FD, FC, and FDC for multilinguals compared to the monolinguals in several fiber bundles involved in domain-general cognitive processes, including the right CST, and the fornix. Differences in these seem to be mostly related to changes in the FC. Importantly, similar fiber bundles have been shown to exhibit reduced FA in simultaneous interpreters compared to multilinguals (Elmer et al., 2011; see details of the study described above). The CST and the midbody of the CC contain fibers related to motor control. The changes observed in these bundles may be related to adaptations supporting speech-articulation and sensory-motor planning (sound-to-motor mapping), fundamental in language production and perception. Thus, these may be the result of long-term adaptations to the greater demands of mapping sounds to the multiple articulation gestures in all languages spoken by the multilingual (starting with the acquisition and usage of a first second language). In relation to Elmer et al. (2011), our results suggest that it is not merely the act of interpreting that leads to changes in sensory-motor mapping mechanisms and interhemispheric communication pathways, but rather the ample opportunities to engage in multilanguage contexts and the need to exert control in multilinguistic environments.

### Correlation Analyses

Additional correlation analyses showed two main trends. Multilinguals exhibited lower FD values in the IFOF (as well as the UF) compared to the monolinguals, however, generally, these were negatively correlated with L2 performance (*r* values ranging from −0.35 to −0.60), indicating that lower FD values in the IFOF are associated with lower performance in the L2 language tests. These patterns of results may reflect dynamic brain adaptations that not only depend upon individual language experience but also may not be fully captured at a certain point in time (e.g., increases in the FC may be observed before or after changes in the FD), underlying the need for longitudinal studies that document WM changes over time, also noted by other authors (e.g., Pliatsikas, 2020). Worth noting is the observation that the IFOF did not show high/significant correlations with L3 performance, suggesting that IFOF modifications might, at least partly, relate to language age of acquisition.

On the other hand, the changes in the FC of the ILF tract showed a generally greater association with the L3 and L1 proficiency (*r* values ranging from 0.30 to 0.60). It is interesting to note that although subjects showed similar proficiency for L2 and L3, at the time of scanning, L3 was (and is) in more current use. The participants were using primarily L3 daily while L2 only on occasion. (The multilingual showed this pattern of L3 > L2 usage for a *M* of 12.5 yrs) The present data suggest that the FC of the ILF tract is modifiable by changes in the frequency and/or contexts or current language use.

Interestingly, the ILF FC values showed the strongest associations with the native language scores for the monolinguals as well; however, these were mainly negative associations (*r* ranging from −0.03 to −0.50), pointing to lower WM values for monolinguals that had better performance in the native language, and hence needed less effort or control.

Considered together, the patterns of results in the correlation analyses point to potential differences in brain resources used to process languages that are used more versus less at a certain time. Additionally, these results highlight the need for and importance of examining specific language experiences in studies investigating the neuroanatomical effects of multilingual experience.

### Conclusion

This study provides unique evidence of brain WM plasticity associated with peak efficiency expertise in multiple languages. Consistent with recent models of brain adaptations in bilinguals (e.g., the BAPSS and the DRM) that describe bilingualism as a dynamic spectrum influenced by experience-based factors that differentially affect brain structure and function (Deluca et al., 2019), our results reveal complex differences in the architecture of the fiber bundles across groups. Taken together our results suggest the following:

(1)  Specific patterns of structural changes within language pathways. We observe fiber cross-section increases in brain morphology within language pathways, while overall FD decreases in these pathways. It is impossible to determine whether these changes are due to a later or earlier learned second language, however, according to the DRM model, which suggests re-restructuring of language areas with additional second language learning, these results may reflect remnants of short-term re-restructuring in language pathways (predicted by the DRM; Pliatsikas, 2020) related to learning an L3, later in life, and being highly immersed in the later learned language environment.
(2)  Decreases in WM morphology within pathways involved in domain-general executive control functions. These are suggestive of long-term neural adaptations, as a response to increased demand in control processes related to lifelong multilanguage experience. Overall our results suggest differential efficiencies in neural communication between domain-specific language regions and domain-general cognitive processes underlying multilingual language acquisition and use.

### Limitations and Further Directions

One of the main limitations of the study is the number of participants. Limitation in power in MRI studies often leads to inconsistencies in findings (Munson & Hernandez, 2019), as well as limitations in identifying effects of interest. In this sample, we have 80% power to detect language group differences of 1.06 *SD*s via *t* tests and detect correlations of strength 0.50 or stronger. Hence, further research with larger groups is needed for a better understanding of WM structural changes associated with high proficiency in multiple languages.

Another limitation is the lack of a bilingual group, for additional comparisons with the multilingual group. A bilingual group with a similar background would have allowed for a comparison of the additive effects of multilingualism beyond one second language. However, we considered it important to look at differences across multilinguals and the traditionally used monolingual speakers.

While we have interpreted our WM effects as reflecting changes associated with multilingualism, we must acknowledge the homogeneity in the life experiences of this particular immigrant cohort.

The FBA technique does not provide a direct measure of axonal myelination, which has been proposed as the main factor resulting in the changes observed in bilingual studies. However, additional information is needed, such as the use of T1 relaxometry (De Santis et al., 2016), to estimate the fixed-specific myelin content. Additionally, apparent FD is influenced by the fraction of the volume that is occupied by the crossing fibers within one single voxel and needs to be interpreted with caution. The FD measure is confounded by the changes in axonal density, axonal diameter, and membrane permeability, which all influence the FD metric (for additional details, see Raffelt et al., 2017). More research is necessary, using the fiber-specific characterization of WM changes, to better understand micro- and macrostructural changes related to bi/multilinguals practice.

Lastly, many studies have reported WM interhemispheric asymmetries for specific fiber bundles with both studies using the DTI (e.g., de Schotten et al., 2011; Park et al., 2004) and FBA techniques (Arun et al., 2021; Bokde et al., 2001). Although the results remain contradictory, the many asymmetries within the WM of healthy populations which have been reported with both left > right and right > left dominances may play an important role in the interpretation of the results in studies looking at healthy human subjects. Nonetheless, more research is needed to identify the contribution of fiber-specific asymmetries for the specific metrics assessed through WM measures.

## ACKNOWLEDGMENTS

We would like to thank Dr. Costin Tanase for his help in suggesting imaging protocols and data analysis practices, and Sharon Coffey Corina for helping throughout the length of the project. Thanks to Diana Malancea-Malac for helping with the behavioral and MRI data collection and organization. Additional thanks to Charles D. Arnold for advising with statistical analysis. This research was supported by the UC Davis Imaging Research Center Pilot Program.

## FUNDING INFORMATION

David P. Corina, University of California, Davis Imagine Research Center Plot.

## AUTHOR CONTRIBUTIONS

**Ludmila Midrigan-Ciochina**: Conceptualization; Formal Analysis; Project administration; Software; Visualization; Writing – original draft; Writing – review & editing. **Kayla P. Vodacek**: Conceptualization; Writing – review & editing. **Cristina Sewell**: Software; Writing – review & editing. **David P. Corina**: Conceptualization; Funding acquisition; Methodology; Resources; Writing – review & editing.

## DATA AND CODE AVAILABILITY STATEMENT

The data that support the findings of this study are available in the OpenNeuro repository, at https://doi.org/10.18112/openneuro.ds004581.v2.0.1. We followed the recommended data preprocessing and analysis pipelines provided by the authors of MRtrix3 (www.mrtrix.org) for fiber density and cross-section, single tissue CSD; this code is freely available at https://github.com/MRtrix3/mrtrix3/blob/master/docs/fixel_based_analysis/st_fibre_density_cross-section.rst. Additionally, we used the TractSeg, and suggested pipelines, available at https://github.com/MIC-DKFZ/TractSeg/tree/master for the creation of streamlines.

## Supplementary Material



## TECHNICAL TERMS:

Brain restructuring: Brain reorganization through forming new neural pathways throughout life and in response to experiences.
Sequential bilinguals: Bilinguals who have learned a second language later in life, sequential to the native language.
Simultaneous bilinguals: Bilinguals who have learned a second language at birth, simultaneously with the native language.
Constrained spherical deconvolution (CSD): Advanced method of modeling complex brain structures that offers vastly greater detail when mapping complex brain network connections.
The partial volume effect (PVE): An issue with tissue estimation, that arises in volumetric images when more than one tissue type occurs in a voxel.
Fiber orientation distribution (FOD): Images reconstructed from diffusion weighted images; widely used to represent the spatial and orientational distribution of neural fibers.
Tractography: A 3D reconstruction technique used to map the location and direction of white matter bundles and their constituent fibers within the human brain.

## References

[bib1] Abutalebi, J., & Green, D. W. (2016). Neuroimaging of language control in bilinguals: Neural adaptation and reserve. Bilingualism, 19(4), 689–698. 10.1017/S1366728916000225

[bib2] Anderson, J. A. E., Grundy, J. G., De Frutos, J., Barker, R. M., Grady, C., & Bialystok, E. (2018). Effects of bilingualism on white matter integrity in older adults. NeuroImage, 167, 143–150. 10.1016/j.neuroimage.2017.11.038, 29175203 PMC5845836

[bib3] Andersson, J. L. R., & Sotiropoulos, S. N. (2016). An integrated approach to correction for off-resonance effects and subject movement in diffusion MR imaging. NeuroImage, 125, 1063–1078. 10.1016/j.neuroimage.2015.10.019, 26481672 PMC4692656

[bib4] Arun, A. H., Connelly, A., Smith, R. E., & Calamante, F. (2021). Characterisation of white matter asymmetries in the healthy human brain using diffusion MRI fixel-based analysis. NeuroImage, 225, Article 117505. 10.1016/j.neuroimage.2020.117505, 33147511

[bib5] Ashburner, J., & Friston, K. J. (2000). Voxel-based morphometry: The methods. NeuroImage, 11(6), 805–821. 10.1006/nimg.2000.0582, 10860804

[bib6] Basser, P. J., & Pierpaoli, C. (2011). Microstructural and physiological features of tissues elucidated by quantitative-diffusion-tensor MRI. Journal of Magnetic Resonance, 213(2), 560–570. 10.1016/j.jmr.2011.09.022, 22152371

[bib7] Beaulieu, C. (2002). The basis of anisotropic water diffusion in the nervous system: A technical review. NMR in Biomedicine, 15(7–8), 435–455. 10.1002/nbm.782, 12489094

[bib8] Bokde, A. L. W., Tagamets, M.-A., Friedman, R. B., & Horwitz, B. (2001). Functional interactions of the inferior frontal cortex during the processing of words and word-like stimuli. Neuron, 30(2), 609–617. 10.1016/S0896-6273(01)00288-4, 11395018

[bib9] Brauer, J., Anwander, A., Perani, D., & Friederici, A. D. (2013). Dorsal and ventral pathways in language development. Brain and Language, 127(2), 289–295. 10.1016/j.bandl.2013.03.001, 23643035

[bib10] Bubb, E. J., Metzler-Baddeley, C., & Aggleton, J. P. (2018). The cingulum bundle: Anatomy, function, and dysfunction. Neuroscience and Biobehavioral Reviews, 92, 104–127. 10.1016/j.neubiorev.2018.05.008, 29753752 PMC6090091

[bib11] Catani, M., & de Schotten, M. T. (2008). A diffusion tensor imaging tractography atlas for virtual in vivo dissections. Cortex, 44(8), 1105–1132. 10.1016/j.cortex.2008.05.004, 18619589

[bib12] Ciochina, L. M., Boyd, V., Ortega, L. S., Malancea-Malac, D., Midrigan, D., & Corina, D. P. (2020). A representative corpus of the Romanian language: Resources in underrepresented languages. In Proceedings of the 12th Conference on Language Resources and Evaluation (LREC 2020) (pp. 3291–3296). European Language Resources Association.

[bib13] Coggins, P. E., III, Kennedy, T. J., & Armstrong, T. A. (2004). Bilingual corpus callosum variability. Brain and Language, 89(1), 69–75. 10.1016/S0093-934X(03)00299-2, 15010238

[bib14] Cummine, J., & Boliek, C. A. (2013). Understanding white matter integrity stability for bilinguals on language status and reading performance. Brain Structure and Function, 218(2), 595–601. 10.1007/s00429-012-0466-6, 23097036

[bib16] Del Maschio, N., Sulpizio, S., Toti, M., Caprioglio, C., Del Mauro, G., Fedeli, D., & Abutalebi, J. (2020). Second language use rather than second language knowledge relates to changes in white matter microstructure. Journal of Cultural Cognitive Science, 4(2), 165–175. 10.1007/s41809-019-00039-z

[bib15] Deluca, V., Rothman, J., & Pliatsikas, C. (2019). Linguistic immersion and structural effects on the bilingual brain: A longitudinal study. Bilingualism: Language and Cognition, 22(5), 1160–1175. 10.1017/S1366728918000883

[bib17] De Santis, S., Assaf, Y., Jeurissen, B., Jones, D. K., & Roebroeck, A. (2016). T1 relaxometry of crossing fibres in the human brain. NeuroImage, 141, 133–142. 10.1016/j.neuroimage.2016.07.037, 27444568 PMC5035137

[bib18] de Schotten, M. T., Ffytche, D. H., Bizzi, A., Dell’Acqua, F., Allin, M., Walshe, M., Murray, R., Williams, S. C., Murphy, D. G. M., & Catani, M. (2011). Atlasing location, asymmetry and inter-subject variability of white matter tracts in the human brain with MR diffusion tractography. NeuroImage, 54(1), 49–59. 10.1016/j.neuroimage.2010.07.055, 20682348

[bib19] Dhollander, T., Clemente, A., Singh, M., Boonstra, F., Civier, O., Dominguez Duque, J., Egorova, N., Enticott, P., Fuelscher, I., Gajamange, S., Genc, S., Gottlieb, E., Hyde, C., Imms, P., Kelly, C., Kirkovski, M., Kolbe, S., Liang, X., Malhotra, A., … Caeyenberghs, K. (2021). Fixel-based analysis of diffusion MRI: Methods, applications, challenges and opportunities. NeuroImage, 241, Article 118417. 10.1016/j.neuroimage.2021.118417, 34298083

[bib20] Dhollander, T., Raffelt, D., & Connelly, A. (2016, September). Unsupervised 3-tissue response function estimation from single-shell or multi-shell diffusion MR data without a co-registered T1 image [Paper presentation]. ISMRM workshop on breaking the barriers of diffusion MRI, Lisbon, Portugal. https://www.researchgate.net/publication/307863133_Unsupervised_3-tissue_response_function_estimation_from_single-shell_or_multi-shell_diffusion_MR_data_without_a_co-registered_T1_image

[bib21] Elmer, S., Hänggi, J., & Jäncke, L. (2014). Processing demands upon cognitive, linguistic, and articulatory functions promote grey matter plasticity in the adult multilingual brain: Insights from simultaneous interpreters. Cortex, 54(1), 179–189. 10.1016/j.cortex.2014.02.014, 24699036

[bib22] Elmer, S., Hänggi, J., Meyer, M., & Jäncke, L. (2011). Differential language expertise related to white matter architecture in regions subserving sensory-motor coupling, articulation, and interhemispheric transfer. Human Brain Mapping, 32(12), 2064–2074. 10.1002/hbm.21169, 21162044 PMC6870450

[bib23] Farquharson, S., & Tournier, J.-D. (2016). High angular resolution diffusion imaging BT. In W. Van Hecke, L. Emsell, & S. Sunaert (Eds.), Diffusion tensor imaging: A practical handbook (pp. 383–406). Springer. 10.1007/978-1-4939-3118-7_20

[bib24] Friederici, A. D. (2009). Pathways to language: Fiber tracts in the human brain. Trends in Cognitive Science, 13(4), 175–181. 10.1016/j.tics.2009.01.001, 19223226

[bib25] Friederici, A. D. (2012). The cortical language circuit: From auditory perception to sentence comprehension. Trends in Cognitive Sciences, 16(5), 262–268. 10.1016/j.tics.2012.04.001, 22516238

[bib26] Friederici, A. D., & Gierhan, S. M. E. (2013). The language network. Current Opinion in Neurobiology, 23(2), 250–254. 10.1016/j.conb.2012.10.002, 23146876

[bib27] Friederici, A. D., von Cramon, D. Y., & Kotz, S. A. (2007). Role of the corpus callosum in speech comprehension: Interfacing syntax and prosody. Neuron, 53(1), 135–145. 10.1016/j.neuron.2006.11.020, 17196536

[bib28] García-Pentón, L., Pérez Fernández, A., Iturria-Medina, Y., Gillon-Dowens, M., & Carreiras, M. (2014). Anatomical connectivity changes in the bilingual brain. NeuroImage, 84, 495–504. 10.1016/j.neuroimage.2013.08.064, 24018306

[bib29] Giorgio, A., Watkins, K. E., Chadwick, M., James, S., Winmill, L., Douaud, G., De Stefano, N., Matthews, P. M., Smith, S. M., Johansen-Berg, H., & James, A. C. (2010). Longitudinal changes in grey and white matter during adolescence. NeuroImage, 49(1), 94–103. 10.1016/j.neuroimage.2009.08.003, 19679191

[bib30] Gold, B. T., Johnson, N. F., & Powell, D. K. (2013). Lifelong bilingualism contributes to cognitive reserve against white matter integrity declines in aging. Neuropsychologia, 51(13), 2841–2846. 10.1016/j.neuropsychologia.2013.09.037, 24103400 PMC3856701

[bib31] Grundy, J. G., Anderson, J. A. E., & Bialystok, E. (2017). Neural correlates of cognitive processing in monolinguals and bilinguals. Annals of the New York Academy of Sciences, 1396(1), 183–201. 10.1111/nyas.13333, 28415142 PMC5446278

[bib32] Hämäläinen, S., Sairanen, V., Leminen, A., & Lehtonen, M. (2017). Bilingualism modulates the white matter structure of language-related pathways. NeuroImage, 152, 249–257. 10.1016/j.neuroimage.2017.02.081, 28263923

[bib33] Hickok, G., & Poeppel, D. (2004). Dorsal and ventral streams: A framework for understanding aspects of the functional anatomy of language. Cognition, 92(1–2), 67–99. 10.1016/j.cognition.2003.10.011, 15037127

[bib34] Hickok, G., & Poeppel, D. (2007). The cortical organization of speech understanding. Nature Reviews Neuroscience, 8(5), 393–402. 10.1038/nrn2113, 17431404

[bib35] Ivanova, I., Salmon, D. P., & Gollan, T. H. (2013). Multilingual naming test in Alzheimer’s disease: Clues to the origin of naming impairments. Journal of the International Neuropsychological Society, 19(3), 272–283. 10.1017/S1355617712001282, 23298442 PMC4356120

[bib36] Jeurissen, B., Leemans, A., Tournier, J.-D., Jones, D. K., & Sijbers, J. (2013). Investigating the prevalence of complex fiber configurations in white matter tissue with diffusion magnetic resonance imaging. Human Brain Mapping, 34(11), 2747–2766. 10.1002/hbm.22099, 22611035 PMC6870534

[bib37] Jones, D. K., Knösche, T. R., & Turner, R. (2013). White matter integrity, fiber count, and other fallacies: The do’s and don’ts of diffusion MRI. NeuroImage, 73, 239–254. 10.1016/j.neuroimage.2012.06.081, 22846632

[bib38] Jouravlev, O., Mineroff, Z., Blank, I. A., & Fedorenko, E. (2021). The small and efficient language network of polyglots and hyper-polyglots. Cerebral Cortex, 31(1), 62–76. 10.1093/cercor/bhaa205, 32820332 PMC7727365

[bib39] Kovelman, I., Baker, S. A., & Petitto, L.-A. (2008). Bilingual and monolingual brains compared: A functional magnetic resonance imaging investigation of syntactic processing and a possible “neural signature” of bilingualism. Journal of Cognitive Neuroscience, 20(1), 153–169. 10.1162/jocn.2008.20011, 17919083 PMC2643466

[bib40] Kuhl, P. K., Stevenson, J., Corrigan, N. M., van den Bosch, J. J. F., Can, D. D., & Richards, T. (2016). Neuroimaging of the bilingual brain: Structural brain correlates of listening and speaking in a second language. Brain and Language, 162, 1–9. 10.1016/j.bandl.2016.07.004, 27490686

[bib41] Leclercq, D., Duffau, H., Delmaire, C., Capelle, L., Gatignol, P., Ducros, M., Chiras, J., & Lehéricy, S. (2010). Comparison of diffusion tensor imaging tractography of language tracts and intraoperative subcortical stimulations. Journal of Neurosurgery, 112(3), 503–511. 10.3171/2009.8.JNS09558, 19747052

[bib42] Linebarger, M. C., Schwartz, M. F., & Saffran, E. M. (1983). Syntactic processing in agrammatism: A reply to Zurif and Grodzinsky. Cognition, 15(1–3), 215–225. 10.1016/0010-0277(83)90042-2 6686509

[bib43] Luk, G., Bialystok, E., Craik, F. I. M., & Grady, C. L. (2011). Lifelong bilingualism maintains white matter integrity in older adults. Journal of Neuroscience, 31(46), 16808–16813. 10.1523/JNEUROSCI.4563-11.2011, 22090506 PMC3259110

[bib44] Makris, N., Kennedy, D. N., McInerney, S., Sorensen, A. G., Wang, R., Caviness, V. S., Jr., & Pandya, D. N. (2005). Segmentation of subcomponents within the superior longitudinal fascicle in humans: A quantitative, in vivo, DT-MRI study. Cerebral Cortex, 15(6), 854–869. 10.1093/cercor/bhh186, 15590909

[bib45] Mamiya, P. C., Richards, T. L., Coe, B. P., Eichler, E. E., & Kuhl, P. K. (2016). Brain white matter structure and COMT gene are linked to second-language learning in adults. Proceedings of the National Academy of Sciences of the United States of America, 113(26), 7249–7254. 10.1073/pnas.1606602113, 27298360 PMC4932981

[bib46] Mancini, M., Karakuzu, A., Cohen-Adad, J., Cercignani, M., Nichols, T. E., & Stikov, N. (2020). An interactive meta-analysis of MRI biomarkers of myelin. ELife, 9, Article e61523. 10.7554/eLife.61523, 33084576 PMC7647401

[bib47] Mandonnet, E., Nouet, A., Gatignol, P., Capelle, L., & Duffau, H. (2007). Does the left inferior longitudinal fasciculus play a role in language? A brain stimulation study. Brain, 130(3), 623–629. 10.1093/brain/awl361, 17264096

[bib48] Martino, J., Hamer, P. C. D. W., Berger, M. S., Lawton, M. T., Arnold, C. M., de Lucas, E. M., & Duffau, H. (2013). Analysis of the subcomponents and cortical terminations of the perisylvian superior longitudinal fasciculus: A fiber dissection and DTI tractography study. Brain Structure and Function, 218(1), 105–121. 10.1007/s00429-012-0386-5, 22422148

[bib49] Midrigan-Ciochina, L., Vodacek, K. P., Balabhadra, S., & Corina, D. P. (2023). A comparison of structural brain differences in monolingual and highly proficient multilingual speakers. Bilingualism: Language and Cognition, 27(1), 117–127. 10.1017/S1366728923000445

[bib51] Mohades, S. G., Struys, E., Van Schuerbeek, P., Mondt, K., Van De Craen, P., & Luypaert, R. (2012). DTI reveals structural differences in white matter tracts between bilingual and monolingual children. Brain Research, 1435, 72–80. 10.1016/j.brainres.2011.12.005, 22197702

[bib50] Mohades, S. G., Van Schuerbeek, P., Rosseel, Y., Van De Craen, P., Luypaert, R., & Baeken, C. (2015). White-matter development is different in bilingual and monolingual children: A longitudinal DTI study. PLOS ONE, 10(2), Article e0117968. 10.1371/journal.pone.0117968, 25706865 PMC4338107

[bib52] Munson, B. A., & Hernandez, A. E. (2019). Inconsistency of findings due to low power: A structural MRI study of bilingualism. Brain and Language, 195, Article 104642. 10.1016/j.bandl.2019.104642, 31238122 PMC8590736

[bib53] Nichols, T. E., & Holmes, A. P. (2002). Nonparametric permutation tests for functional neuroimaging: A primer with examples. Human Brain Mapping, 15(1), 1–25. 10.1002/hbm.1058, 11747097 PMC6871862

[bib54] Nosarti, C., Rushe, T. M., Woodruff, P. W. R., Stewart, A. L., Rifkin, L., & Murray, R. M. (2004). Corpus callosum size and very preterm birth: Relationship to neuropsychological outcome. Brain, 127(9), 2080–2089. 10.1093/brain/awh230, 15289268

[bib55] Oishi, K., Faria, A., Jiang, H., Li, X., Akhter, K., Zhang, J., Hsu, J. T., Miller, M. I., van Zijl, P. C. M., Albert, M., Lyketsos, C. G., Woods, R., Toga, A. W., Pike, G. B., Rosa-Neto, P., Evans, A., Mazziotta, J., & Mori, S. (2009). Atlas-based whole brain white matter analysis using large deformation diffeomorphic metric mapping: Application to normal elderly and Alzheimer’s disease participants. NeuroImage, 46(2), 486–499. 10.1016/j.neuroimage.2009.01.002, 19385016 PMC2885858

[bib56] Oldfield, R. C. (1971). The assessment and analysis of handedness: The Edinburgh inventory. Neuropsychologia, 9(1), 97–113. 10.1016/0028-3932(71)90067-4, 5146491

[bib57] Papadakis, N. G., Martin, K. M., Mustafa, M. H., Wilkinson, I. D., Griffiths, P. D., Huang, C. L.-H., & Woodruff, P. W. R. (2002). Study of the effect of CSF suppression on white matter diffusion anisotropy mapping of healthy human brain. Magnetic Resonance in Medicine, 48(2), 394–398. 10.1002/mrm.10204, 12210950

[bib58] Park, H.-J., Westin, C.-F., Kubicki, M., Maier, S. E., Niznikiewicz, M., Baer, A., Frumin, M., Kikinis, R., Jolesz, F. A., McCarley, R. W., & Shenton, M. E. (2004). White matter hemisphere asymmetries in healthy subjects and in schizophrenia: A diffusion tensor MRI study. NeuroImage, 23(1), 213–223. 10.1016/j.neuroimage.2004.04.036, 15325368 PMC2794419

[bib59] Pliatsikas, C. (2019). Multilingualism and brain plasticity. In J. W. Schwieter & M. Paradis (Eds.), The handbook of the neuroscience of multilingualism (pp. 230–251). Wiley. 10.1002/9781119387725.ch11

[bib60] Pliatsikas, C. (2020). Understanding structural plasticity in the bilingual brain: The dynamic restructuring model. Bilingualism: Language and Cognition, 23(2), 459–471. 10.1017/S1366728919000130

[bib61] Pliatsikas, C., DeLuca, V., & Voits, T. (2020). The many shades of bilingualism: Language experiences modulate adaptations in brain structure. Language Learning, 70(S2), 133–149. 10.1111/lang.12386

[bib62] Pliatsikas, C., & Luk, G. (2016). Executive control in bilinguals: A concise review on fMRI studies. Bilingualism: Language and Cognition, 19(4), 699–705. 10.1017/S1366728916000249

[bib63] Pliatsikas, C., Moschopoulou, E., & Saddy, J. D. (2015). The effects of bilingualism on the white matter structure of the brain. Proceedings of the National Academy of Sciences of the United States of America, 112(5), 1334–1337. 10.1073/pnas.1414183112, 25583505 PMC4321232

[bib64] Pollmann, S., Maertens, M., von Cramon, D. Y., Lepsien, J., & Hugdahl, K. (2002). Dichotic listening in patients with splenial and nonsplenial callosal lesions. Neuropsychology, 16(1), 56–64. 10.1037/0894-4105.16.1.56, 11858226

[bib65] Qi, Z., Han, M., Garel, K., San Chen, E., & Gabrieli, J. D. E. (2015). White-matter structure in the right hemisphere predicts Mandarin Chinese learning success. Journal of Neurolinguistics, 33, 14–28. 10.1016/j.jneuroling.2014.08.004

[bib66] R Core Team. (2020). R: A language and environment for statistical computing (Version 4.0.3) [Software]. R Foundation for Statistical Computing. https://www.R-project.org/

[bib67] Raffelt, D. A., Smith, R. E., Ridgway, G. R., Tournier, J.-D., Vaughan, D. N., Rose, S., Henderson, R., & Connelly, A. (2015). Connectivity-based fixel enhancement: Whole-brain statistical analysis of diffusion MRI measures in the presence of crossing fibres. NeuroImage, 117, 40–55. 10.1016/j.neuroimage.2015.05.039, 26004503 PMC4528070

[bib69] Raffelt, D., Tournier, J.-D., Fripp, J., Crozier, S., Connelly, A., & Salvado, O. (2011). Symmetric diffeomorphic registration of fibre orientation distributions. NeuroImage, 56(3), 1171–1180. 10.1016/j.neuroimage.2011.02.014, 21316463

[bib70] Raffelt, D., Tournier, J.-D., Rose, S., Ridgway, G. R., Henderson, R., Crozier, S., Salvado, O., & Connelly, A. (2012). Apparent fibre density: A novel measure for the analysis of diffusion-weighted magnetic resonance images. NeuroImage, 59(4), 3976–3994. 10.1016/j.neuroimage.2011.10.045, 22036682

[bib68] Raffelt, D. A., Tournier, J.-D., Smith, R. E., Vaughan, D. N., Jackson, G., Ridgway, G. R., & Connelly, A. (2017). Investigating white matter fibre density and morphology using fixel-based analysis. NeuroImage, 144(A), 58–73. 10.1016/j.neuroimage.2016.09.029, 27639350 PMC5182031

[bib71] Rahmani, F., Sobhani, S., & Aarabi, M. H. (2017). Sequential language learning and language immersion in bilingualism: Diffusion MRI connectometry reveals microstructural evidence. Experimental Brain Research, 235(10), 2935–2945. 10.1007/s00221-017-5029-x, 28702836

[bib72] Schlegel, A. A., Rudelson, J. J., & Tse, P. U. (2012). White matter structure changes as adults learn a second language. Journal of Cognitive Neuroscience, 24(8), 1664–1670. 10.1162/jocn_a_00240, 22571459

[bib73] Schwarz, C. G., Reid, R. I., Gunter, J. L., Senjem, M. L., Przybelski, S. A., Zuk, S. M., Whitwell, J. L., Vemuri, P., Josephs, K. A., Kantarci, K., Thompson, P. M., Petersen, R. C., Jack, C. R., Jr., & Alzheimer’s Disease Neuroimaging Initiative. (2014). Improved DTI registration allows voxel-based analysis that outperforms tract-based spatial statistics. NeuroImage, 94, 65–78. 10.1016/j.neuroimage.2014.03.026, 24650605 PMC4137565

[bib74] Seo, R., Stocco, A., & Prat, C. S. (2018). The bilingual language network: Differential involvement of anterior cingulate, basal ganglia and prefrontal cortex in preparation, monitoring, and execution. NeuroImage, 174, 44–56. 10.1016/j.neuroimage.2018.02.010, 29486320

[bib75] Shipley, W. C. (1940). A self-administering scale for measuring intellectual impairment and deterioration. Journal of Psychology: Interdisciplinary and Applied, 9(2), 371–377. 10.1080/00223980.1940.9917704

[bib76] Singh, N. C., Rajan, A., Malagi, A., Ramanujan, K., Canini, M., Pasquale, E., Della Rosa, P. A., Raghunathan, P., Weekes, B. S., & Abutalebi, J. (2018). Microstructural anatomical differences between bilinguals and monolinguals. Bilingualism: Language and Cognition, 21(5), 995–1008. 10.1017/S1366728917000438

[bib77] Smith, R. E., Tournier, J.-D., Calamante, F., & Connelly, A. (2013). SIFT: Spherical-deconvolution informed filtering of tractograms. NeuroImage, 67, 298–312. 10.1016/j.neuroimage.2012.11.049, 23238430

[bib78] Smith, R. E., Tournier, J.-D., Calamante, F., & Connelly, A. (2015). SIFT2: Enabling dense quantitative assessment of brain white matter connectivity using streamlines tractography. NeuroImage, 119, 338–351. 10.1016/j.neuroimage.2015.06.092, 26163802

[bib79] Smith, S. M., Jenkinson, M., Woolrich, M. W., Beckmann, C. F., Behrens, T. E. J., Johansen-Berg, H., Bannister, P. R., De Luca, M., Drobnjak, I., Flitney, D. E., Niazy, R. K., Saunders, J., Vickers, J., Zhang, Y., De Stefano, N., Brady, J. M., & Matthews, P. M. (2004). Advances in functional and structural MR image analysis and implementation as FSL. NeuroImage, 23(S1), S208–S219. 10.1016/j.neuroimage.2004.07.051, 15501092

[bib80] Tournier, J.-D., Calamante, F., & Connelly, A. (2007). Robust determination of the fibre orientation distribution in diffusion MRI: Non-negativity constrained super-resolved spherical deconvolution. NeuroImage, 35(4), 1459–1472. 10.1016/j.neuroimage.2007.02.016, 17379540

[bib81] Tournier, J.-D., Calamante, F., Gadian, D. G., & Connelly, A. (2004). Direct estimation of the fiber orientation density function from diffusion-weighted MRI data using spherical deconvolution. NeuroImage, 23(3), 1176–1185. 10.1016/j.neuroimage.2004.07.037, 15528117

[bib82] Tournier, J.-D., Mori, S., & Leemans, A. (2011). Diffusion tensor imaging and beyond. Magnetic Resonance in Medicine, 65(6), 1532–1556. 10.1002/mrm.22924, 21469191 PMC3366862

[bib83] Tournier, J.-D., Smith, R., Raffelt, D., Tabbara, R., Dhollander, T., Pietsch, M., Christiaens, D., Jeurissen, B., Yeh, C.-H., & Connelly, A. (2019). MRtrix3: A fast, flexible and open software framework for medical image processing and visualisation. NeuroImage, 202, Article 116137. 10.1016/j.neuroimage.2019.116137, 31473352

[bib84] Tremblay, P., & Dick, A. S. (2016). Broca and Wernicke are dead, or moving past the classic model of language neurobiology. Brain and Language, 162, 60–71. 10.1016/j.bandl.2016.08.004, 27584714

[bib85] Tustison, N. J., Avants, B. B., Lin, Z., Feng, X., Cullen, N., Mata, J. F., Flors, L., Gee, J. C., Altes, T. A., Mugler, J. P., III, & Qing, K. (2019). Convolutional neural networks with template-based data augmentation for functional lung image quantification. Academic Radiology, 26(3), 412–423. 10.1016/j.acra.2018.08.003, 30195415 PMC6397788

[bib86] Veraart, J., Novikov, D. S., Christiaens, D., Ades-Aron, B., Sijbers, J., & Fieremans, E. (2016). Denoising of diffusion MRI using random matrix theory. NeuroImage, 142, 394–406. 10.1016/j.neuroimage.2016.08.016, 27523449 PMC5159209

[bib87] Wakana, S., Jiang, H., Nagae-Poetscher, L. M., van Zijl, P. C. M., & Mori, S. (2004). Fiber tract-based atlas of human white matter anatomy. Radiology, 230(1), 77–87. 10.1148/radiol.2301021640, 14645885

[bib88] Wasserthal, J., Neher, P. F., & Maier-Hein, K. H. (2018). Tract orientation mapping for bundle-specific tractography. In A. Frangi, J. Schnabel, C. Davatzikos, C. Alberola-López, & G. Fichtinger (Eds.), Medical image computing and computer assisted intervention (MICCAI 2018). Springer. 10.1007/978-3-030-00931-1_5

