# Supplementary material for: A Comparison of White Matter Brain Differences in Monolingual and Highly Proficient Multilingual Speakers
Source: Neurobiol Lang (Camb). 2024 Jun 3;5(2):497–527. doi: 10.1162/nol_a_00144 (PMC11192512; doi:10.1162/nol_a_00144)
Supplement: Supplementary file 1 [file nol-5-2-497-s001.pdf]

Suplementary Materials

BEHAVIORAL DATA

Table 1. Participants' Background Summary

Participants' Background Summary

Monolingual Participants

P	*SeSp*	*SeSs*	*%DU*
1	0.7	-0.3	100
2	-.23	-0.3	100
3	-1.1	-0.3	100
4	-1.1	-0.3	100
5	1.5	-0.3	100
6	-1.1	-1.1	100
7	-1.1	-0.2	100
8	2.5	.66	100
9	0.6	-0.3	100
10	1.6	-0.3	100
11	0.6	-0.3	100
12	1.5	-1.1	100
13	0.6	-0.3	100
14	1.5	0.6	100
15	-1.1	0.6	100

Multilingual Participants

P	*SeSp*	*SeSs*	*Romanian (native)*		*Russian*		*English*	
			%DU	*Env*	*%DU*	*Env*	*~%DU*	*Env*
16	0.6	-1.5	40	home, church	10	family	50	school, work
17	-1.5	-0.3	40	home, church	5	movies	55	school, work
18	-1.5	-0.3	40	home	5	news, movies	55	school, work
19	0.6	0.9	60	home, church	10	family	30	work, church, home
20	0.6	0.9	60	home, church	10	work, friends	30	work, church, home
21	0.6	-1.9	30	home, church	20	family, friends	50	work, church, home
22	0.6	-.9	45	home, church	5	spouse family	50	work, home, school
23	0.6	0.6	40	home, church	5	vlogs online	55	work, church
24	-0.12	-0.3	40	home, church	20	spouse, vlogs	40	work, home
25	-0.12	-0.1	30	home, church	10	spouse family	60	church, home
26	-0.12	0.9	50	home	20	family, work	30	work
27	-0.12	-1.9	40	home, church	10	friends	50	work, church
28	-0.12	-1.5	40	home	3	spouse family	57	work, school
29	0.6	0.9	30	home	10	family, friends	60	work, home, school
30	0.6	-1.5	40	home	1	vlogs	59	work, home, school

Table 1. Participants' Background Description. P: participant number; SeSp: socioeconomic status (parent), standardized; SeSp: socioeconomic status (participant), standardized; %DU ; approximate daily use in (in percent); Note that all monolinguals participants speak only English, while multilinguals speak Romanian natively, Russian as an early learned L2, and English as a later learned L3.

PROFICIENCY ASSESSMENT TESTS

Language Production and Vocabulary Assessment Results

The mean for the percentage of error for the MINT was 6.9% (SD= 3.2%) for naming in native English for the monolingual group. The percentage error for the multilinguals was 5.4% (SD = 7.2 %) for L1, 25.4% (SD = 15.4%) for L2, and 24.7% (SD = 6.9%) for L3 (see Table 2). Two sample t-tests were carried out to compare proficiency between the groups for the native language. The difference between the L1 monolinguals and L1 multilinguals ($t_{(28)} = 1.0$ $p < 0.3$; 95% CI, 1.85 to 0.65) was not significant. Paired sample t-tests were computed to assess the accuracy of the MINT, and further investigate differences in proficiency within the multilingual group between the languages.

Table 2. Percentage Error for Proficiency Measures

Percentage Error for the Proficiency Measures				
	MINT	SHIPLEY	GAT	Avg. Proficiency
	Perc. of Error	Perc. of Error	Perc. of Error	Perc. of Error
L1(mono)	6.9 (SD 3.2)	16.8 (SD 5.9)	13.9 (SD 8.7)	11.8 (SD 5.3)
L1(multi)	5.4 (SD 7.2)	12.8 (SD 7)	10.7 (SD 8.6)	10.0 (SD 5.3)
L2(multi)	25.4 (SD 15.4)	16.8 (SD 12.6)	18.2 (SD 7.5)	19.4 (8.5)
L3(multi)	24.7(SD 6.9)	24.5 (SD 18.5)	11.6 (SD 8.2)	18.4 (9.6)

Table 2. Description: Note: Mono: monolingual; Multi: multilingual; Perc. of Error: percentage of error; Avg. Proficiency: average proficiency

The differences between the L1 and L2 scores ($t_{(14)} = 7.2$, $p < 0.001$; 95% CI, 4.45 to 8.21) and the L1 and L3 ($t_{(14)} = 4.04$, $p < 0.001$; 95% CI, 2.87 to 9.38) were significant. There was no difference in scores between the L2 and L3 ($t_{(14)} = 0.1$, $p = 0.9$; 95% CI, 3.3 to 2.9) (see Table 2).

Semantic and Vocabulary Knowledge Test Results

The mean for the percentage of error for the Semantic and Vocabulary Knowledge Test was 16.8% (SD= 5.9%) for the monolingual group (see Table 2). The percentage errors for the multilingual group were 12.8% (SD = 7%) for the native L1, 16.8% (SD = 12.6%) for the L2, and 24.5 % (SD = 18.5 %) for the L3. A two-sample t-test between the groups showed nonsignificant results ($t_{(28)} = 1.9$, $p = 0.6$; 95% CI, 3.26 to 0.06). Paired sample t-tests showed statistically significant differences between L1 and L3 scores ($t_{(14)} = 3.6$, $p < 0.01$; 95% CI, 1.85 to 7.48),

while the differences between L1 versus L2 ($t_{(14)} = 1.3$, p=0.2; 95% CI, 0.95 to 4.15) and L2 versus L3($t_{(14)} = 1.7$, p=0.1; 95% CI, -0.72 to 6.85) were not significant (see Table 3).

Grammatical Assessment Test Results

The mean percentage error produced in the GAT was 13.9 % (SD = 8.7 %) for the monolinguals. For the multilingual group, the percentage errors were10.7% (SD = 8.6%) for the test in L1, 18.2% (SD = 7.5%) for the L2, and 11.6 % (SD = 8.2%) for the L3 (see Table 2). A two-sample t-test showed insignificant results between the two groups for the grammar test ($t_{(28)}$ = 0.9, p = 0.4; 95% CI, 5.7 to 2.24). Paired t-tests revealed differences between the scores in L1 versus L2 ($t_{(15)}$ = 3.2, p= 0.01; 95% CI, 1.78 to 9.14), and for L1 versus L3 ($t_{(14)}$ = 3.2; p = 0.01; 95% CI,7.9 to 1.6) and no differences between the scores for L2 versus L3 ($t_{(14)}$ = 0.5, p = 0.7; 95% CI, 2.44 to 3.78) (see Table 3).

Table 3. Proficiency Measures Statistic Testing Results

Proficiency Data Statistics												
	MINT			***SHIPLEY***			***GAT***			***Avg. Proficiency***		
	p-value	*t stat.*	*CI (95%)*	*p-value*	*t stat*	*CI (95%)*	*p-value*	*t stat.*	*CI (95%)*	*p-value*	*t stat.*	*CI (95%)*
L1(mono) > L1(multi)	<0.4	-1	-1.85 to 0.65	< 0.6	-1.9	-3.26 to 0.06	< 0.4	0.9	5.7 to 2.24	<0.3	-1.05	-2.56 to 0.83
L1 (multi) > L2 (multi)	< 0.001*	7.2	4.45 to 8.21	< 0.2	1.3	-0.95 to 4.15	< 0.01*	3.2	1.78 to 9.14	<0.001*	4.4	2.3 to 6.63
L1 (multi) > L3 (multi)	< 0.001*	4.04	2.87 to 9.38	<0.01*	3.6	1.85 to 7.48	< 0.6	0.5	-2.4 to 3.7	<0.001*	4.2	1.87 to 5.76
L2 (multi) > L3 (multi)	< 0.9	-0.1	-3.3 to 2.9	< 0.1	1.7	-0.72 to 6.85	< 0.1	0.5	2.44 to 3.78	< 0.6	-0.5	3.3 to 2.01

Table 3. Description: P-values were FDR corrected for the number of tests. Note: Mono: monolingual; Multi: multilingual; GAT: Grammar test, MINT: Multilingual Naming Tests; Shipley: Self-Administering Scale for Measuring Intellectual Impairment and Deterioration, Lang. Use: language use

We have additionally looked at whether the sentences were considered grammatical or ungrammatical depending on whether the grammatical errors translated across languages or not. A comparison between the average rating for each sentence that had a cross-language versus a language-dependent error was computed. The paired two-sample t-tests were not significant for this comparison (see Figure 1). However, our sample had few data points (30 sentences per language), for a thorough comparison, and further analysis is needed for conclusive results.

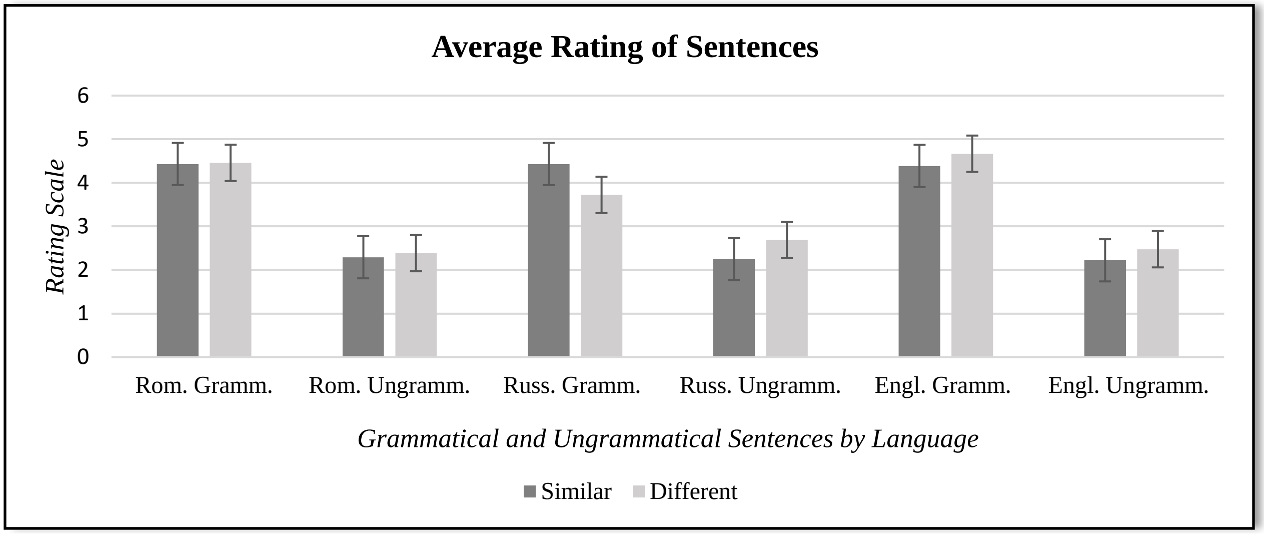

Figure 1. Average Rating of Sentences of Ungrammaticality Scores

Figure 1. Description: Similar – sentence construction with grammatical errors that translated across languages on a scale of 1(ungrammatical) to 5 (grammatical). Different sentence constructions with grammatical errors that did not translate across languages. Rom: Romanian; Russ: Russian; Engl: English. Gramm: Grammatical sentence types; Ungramm: Ungrammatical sentence types.

Average Proficiency Results

All Language proficiency tests were conducted on three different occasions (one for each language) for multilinguals and one single visit for the monolinguals. The language order was randomized across the multilingual participants.

The Development of the Semantic and Vocabulary Knowledge Test.

a. History of the Test

Additionally, the vocabulary section of the *Self-Administering Scale for Measuring Intellectual Impairment and Deterioration Test*, (also called Shipley Test in various studies) was used to assess the semantic and vocabulary

knowledge of our participants. The original test (Shipley, 1940) was developed to assess vocabulary deterioration and abstract thinking and it has been widely used to assess the intellectual ability and vocabulary of clinical as well as normal populations (e.g. Harnish et al., 1994; Martin et al., 1979). For this study, the vocabulary part of the original Shipley was used. This section included 40 items.

b. Adaptation to our study

The English version of the *Self-Administering Scale for Measuring Intellectual Impairment and Deterioration* (a.k.a. Shipley Tests) was translated into Romanian and Russian. The vocabulary items were matched as closely as possible in meaning and difficulty. The normed frequency distributions of each of the items were computed in all three languages. First, word frequencies were computed from the Corpus of Contemporary American English (COCA, approximately 560,000,000 tokens) for each item in the original English test. Second, translation equivalents in Romanian and Russian (a few potential names for each item) were found. Normed frequencies were computed for each translated item in Romanian and Russian from two large corpora. Romanian item frequencies were extracted from the Balanced Corpus of Romanian Language, containing approximately 5,500,000 tokens (Ciochina et al., 2020), and in Russian, from Wikipedia text extracted from the Wiki dump, approximately 442,400,562 tokens. The final list of potential equivalent items was then created based on the frequency distributions obtained from each corpus using tools from the Natural Language Tool Kit, in Python (Bird et al., 2009). The selected words were then judged on a continuous scale from easy to difficult, by two university professors of the Russian language - native Russian speakers (one from Moldova and one from Russia) and two native language speakers of Romanian (both from Romania). This was done to ensure that the tests were matched across languages in terms of increasing difficulty with each succeeding item. Some items, though appearing with lower frequency in the corpora, were judged to be “easy” by the native speakers, whereas others, having high frequency were taught to be “harder”, thus, were replaced by appropriate words similar in meaning and frequency with the English version. Also, many words, especially towards the end of the test appeared with zero frequency in the Russian and Romanian corpora though were considered good matches for the English words. It is also worth mentioning that Romanian, as well as Russian, have a very rich system of declension and each of the lexical items was selected to have similar word forms (e.g. suffixes, case, number, etc.) across the options for each item in order to avoid potential identification of the correct answer. For a complete list of all items used in the *Semantic and Vocabulary Knowledge Test* in all three languages with their respective normed frequencies, see Table 3.

The Development of the Adapted MiNT Test

a. History of the Test

For the assessment of production proficiency and vocabulary knowledge, the participants were asked to complete the Multilingual Naming Test (MiNT; Ivanova et al., 2013). The original test consisted of 68 black-and-white line drawing images from different sources, an adaptation of a 32-image set first used by Ivanova, Salmon & Gollan 2012, for Spanish speakers, was used for this study. The items in the images were selected to represent words of increased difficulty with presentation order. The participants were asked to name 32 black and white drawings of objects as the one in Figure 2.

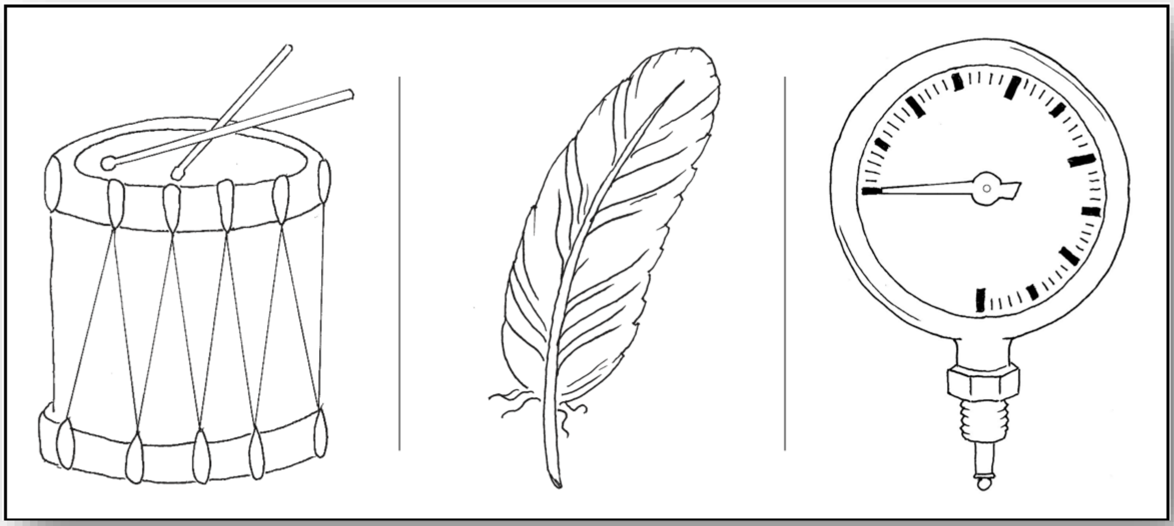

Figure 2. MINT Images with Increased Difficulty

Figure 2. Description: Images number 1 – easy (left), number 15 – medium difficulty (middle) and number 32 – difficult (right).

b. Adaptation to our study

Multilingual participants were asked to name the images in Romanian, Russian, and English, respectively on three different occasions. The language order was randomized across participants. The word frequency associated with each of the images was assessed in Romanian and Russian, to represent decreased frequency with presentation order using the same corpora as for the Shipley test (see above). The same set of images as the original used by

Ivanova, Salmon & Gollan 2013 were found appropriate for the two additional languages. One image was excluded from all languages since it had no specific equivalent name in Romanian (i.e. the image showing a "plug"). Similarly, for two image items more than one name was accepted as the correct answer in Romanian (i.e., the equivalent for 'witch' in Romanian is both 'baba Iaga' (Moldovan dialect) or 'vrăjitoare'; the word 'king' can be translated as both 'rege' and 'împarat').

The Development of the GAT test

a. History of the Test

The Grammatical Assessment Test (GAT) was constructed following a similar methodology as in Linebarger et al., 1983, originally developed to assess comprehension failures in agrammatic aphasics (Linebarger et al., 1983).

b. Adaptation to our study

A new set of sentences for each of the languages (including the English version) was created, specifically designed to assess grammatical judgment and sentence comprehension of Romanian, Russian, and English (namely, 72 sentences for each language). Half of the sentences (i.e., 36) were syntactically well-formed, and 36 were systematically ill-formed - representing 12 types of grammatical rule violations. Seven of the types were violations that translated across all three language structures (Romanian, Russian, and English); whereas the other five were language-dependent.

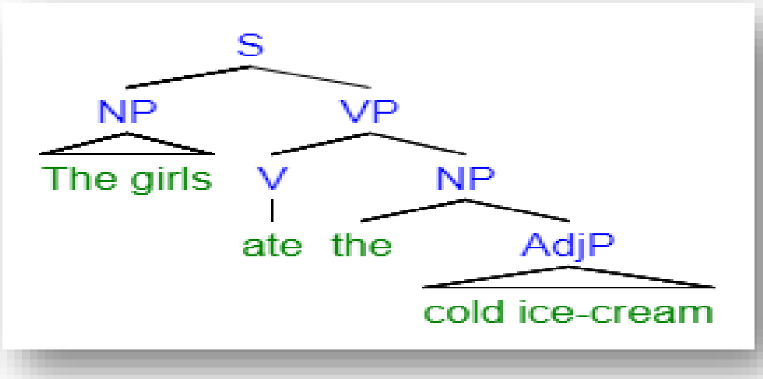

Figure 3. Example of Sentence Construction for the GAT

Figure 3. Description: Sentence tree example generated with the http://mshang.ca/syntree/. Note: S: sentence; NP: noun phrase; VP: verb phrase; AdjP: adjective phrase.

The syntactic structure of all sentences followed the same construction (see Figure 3), containing a noun phrase, a verb phrase, and an adjunct or modifier phrase. Six sentences for each type of sentence construction, three grammatical and three ungrammatical, were presented in the test in a randomized order from participant to participant, with no more than four consecutive sentences being grammatical or ungrammatical. Participants were asked to judge whether the sentence seemed grammatical or ungrammatical to them on a scale of 1 to 5. The sentence order was pseudo-randomized to create three versions of the test for each language, and administered to the participants in a randomized order. Below is an example of a sentence in each language for one of the grammatical construction types used in the test. The ungrammaticality of this type of construction comes from the fact that the intransitive verb is followed by a direct object. Table 4, lists examples of sentences in each language, for each grammatical error type.

Table 4. Shipley Adapted

**English**		**Russian**		**Romanian**	
List of Items	*Normed Freq.*	*List of Items*	*Normed Freq.*	*List of Items*	*Normed Freq.*
TALK	33.08	ГОВОРИТЬ	9.94	VORBI	7.53
PERMIT	19.42	ПОЗВОЛИТЬ	3.21	PERMITE	3.98
PARDON	4.73	ПРОСТИТЬ	2.26	PARDON	3.3
COUCH	4.12	ДИВАН	1.46	CANAPEA	2.27
REMEMBER	2.21	ПОМНИТЬ	0.73	AMINTI	1.65
TUMBLE	2.07	СВАЛИТЬ	0.72	POTICNI	0.97
HIDEOUS	2.03	УЖАСНО	0.57	ÎNGROZITOR	0.84
CORDIAL	1.38	ДРУЖЕЛЮБНЫЙ	0.52	PRIETENOS	0.81
EVIDENT	0.99	ОЧЕВИДНЫЙ	0.44	APARENT	0.81
IMPOSTOR	0.79	САМОЗВАНЕЦ	0.29	ȘARLATAN	0.76
MERIT	0.6	ЗАСЛУГИ	0.18	MERIT	0.62
FASCINATE	0.52	ПРИВЛЕЧЬ	0.11	FERMECA	0.54
INDICATE	0.49	УКАЗАТЬ	0.1	SEMNIFICA	0.49
IGNORANT	0.35	БЕЗГРАМОТНЫЙ	0.1	IGNORANT	0.43
FORTIFY	0.31	УСИЛИВАТЬ	0.09	FORTIFICA	0.35
RENOWN	0.27	СЛАВА	0.07	RENUME	0.32
NARRATE	0.18	ПОВЕСТВОВАТЬ	0.06	RELATA	0.3
MASSIVE	0.12	ОГРОМНЫЙ	0.05	ENORM	0.27
HILARITY	0.11	ВЕСЕЛОСТЬ	0.04	VESELIE	0.24
SMIRCHED	0.1	ГРЯЗНЫЙ	0.03	MÂNJI	0.24
SQUANDER	0.09	РАЗБАЗАРИВАТЬ	0.01	RISIPI	0.22
CAPTION	0.08	ТИТУЛ	0.01	DENUMIRE	0.19
FACILITATE	0.07	СОДЕЙСТВИЯ	0.01	ÎNLESNI	0.19
JOCOSE	0.07	ШУТЛИВЫЙ	0.01	JUCĂUȘ	0.19
APPRISE	0.06	ИЗВЕЩЯТЬ	0.01	AVERITIZA	0.16

RUE	0.06	РАСКАИВАТЬСЯ	0	REGRETA	0.14
DENIZEN	0.06	АБИТАТЕЛЬ	0	LOCĂTAR	0.11
DIVEST	0.05	ЛИШАТЬ	0	DESPUIA	0.11
AMULET	0.05	АМУЛЕТ	0	TALISMAN	0.11
INEXORABLE	0.04	НЕУМОЛИМЫЙ	0	NEÂNDUPLECAT	0.08
SERRATED	0.04	ЗАЗУБРЕННЫЙ	0	ZIMŢAT	0.08
LISSOM	0.03	ГИБКИЙ	0	MLĂDIOS	0.08
MOLLIFY	0.03	УМИРОТВОРИТЬ	0	DOMOLI	0.05
PLAGIARIZE	0.02	ПЛАГАТОРСТВО	0	PLAGIA	0.05
ORIFICE	0.02	ОТВЕРСТИЕ	0	ORIFICIU	0.05
QUERULOUS	0.01	ВОРЧЛИВЫЙ	0	PLÂNGĂREŢ	0.03
PARIAH	0.01	ПАРИЯ	0	IZGONIT	0.03
ABET	0	ПОДСТРЕКАТЬ	0	SPRIJINI	0.03
TEMERITY	0	БЕЗРАССУДСТВО	0	ÎNDRĂZNEALĂ	0
PRISTINE	0	БЕЗУПРЕЧНЫЙ	0	IMPECABIL	0

Table 4. List of all items used in the Semantic and Vocabulary Knowledge Test in all three languages with their respective normed frequencies.

Table 5. Grammatical Assessment Test

Grammatical sentence Type	Ungrammaticality	Example in English, Romanian, and Russian
Type I	Ungrammaticality comes from the fact that the intransitive verb is followed by a direct object:	* James went the campus coffee shop.<br>James went to the campus coffee shop.<br><br>* Anca a venit casa bunicilor<br>Anca a venit la casa bunicilor.<br><br>* Девушка спала диване<br>Девушка спала на диване
Type II	Ungrammaticality comes from the position of the empty NP elements in the sentences.	* That day was promised to be given the test _<br>The test was promised to be given that day.<br><br>* Aceste lucrări urmează să finiseze Maria_<br>Maria urmează să finiseze aceste lucrări.<br><br>* Эту роль, корее всего Райан сыграет_<br>Скорее всего, Райан сыграет эту роль
Type III	Ungrammaticality comes from the fact that the specifier of an NP is fronted by Wh-movement; the head noun of the NP is left behind. The fronted material is interpreted as an NP with the ellipsis of the head.	* Which good did you see friends at the party?<br>Which good friends did you see at the party?<br><br>* Câţi ai invitat prieteni la ziua de naştere?<br>Câţi prieteni ai invitat la ziua de naştere?<br><br>* Какие интересные вы услышали истории в детстве?<br>Какие интересные истории вы услышали в детстве?
Type IV	Ungrammaticality comes from the fact that the gap is absent in the relative clause.	* John knew the poems that she assigned the homework.<br>John knew the poems that she assigned.<br><br>* Vadim nu mi-a adus cartea pe care i-am dat caietul.<br>Vadim nu mi-a adus cartea pe care i-am dat-o.<br><br>* Вы не убирали комнату которую я обычно убираю комнату

		Вы не убрали комнату, которую я обычно убираю?
Type V	Ungrammaticality comes from the violation of phrasal structures (NP – NP NP vs NP – NP PP).	* The newspaper this boutique is from yesterday.<br>The newspaper this boutique sells, is from yesterday.<br><br>* Cartea prietenul meu e foarte interesantă.<br>Cartea pentru prietenul meu e foarte interesantă.<br><br>* Машина мой друг очень чиста.<br>Машина для моего друга очень чиста
Type VI	Ungrammaticality comes from the violation of gender, number or person, or both number and person).	* The neighbors herself were very surprised!<br>The neighbors themselves were very surprised!<br><br>* Ei înşivă vor regreta pierderile suferite.<br>Ei înşişi vor regreta pierderile suferite.<br><br>* В эти выходные мы сам не были дома<br>В эти выходные мы сами не были дома
Type VII	Morphological case violations (specifically, the morphological form of the pronoun that is incompatible with the -*agrt* feature required).	* The list had they in the first place<br>The list had them in the first place.<br><br>* Mama pasăre le-a adus şi ţie nişte viermişori.<br>Mama pasăre le-a adus şi lor nişte viermişori.<br><br>* Маленькая девочка принесла мы кошку.<br>Маленькая девочка принесла ему кошку.
Type VIII	English: Particle Movement construction. Ungrammaticality comes from the fact that the preposition is put after rather than before the NP.<br><br>Romanian: Ungrammaticality comes from the violation of the adjective agreement in number and gender with the noun.<br><br>Russian: Ungrammaticality comes from the violation of the required complementizer for the subordinate clause (i.e. interrogative, subjunctive, and indicative).	* Liz ran the stairs down when she heard the noise.<br>Liz ran down the stairs when she heard the noise.<br><br>* Ele au cumpărat cămaşă albastre.<br>Ele au cumpărat cămaşe albastre.<br><br>* Она спросила, чтобы вы потратили столько денег.<br>Она спросила, почему вы потратили столько денег.
Type IX	English: Ungrammaticality comes from either the existence of a copy of the *aux* in its initial position within the yes/no question formation structures or the *aux* is not appropriate with the verb form.<br><br>Romanian: Ungrammaticality comes from *double-case* violation.<br><br>Russian: Ungrammaticality comes from the fact that infinitival clauses do not allow for overt subjects.	* Is the boy is having a good time?<br>Is the boy having a good time?<br><br>* Ilincuţa l-a ajutat pe bunicului să ducă lemne.<br>Ilincuţa l-a ajutat pe bunicul să ducă lemne.<br><br>* Танцору танцевать - это хороший вид спорта.<br>Танцевать - это хороший вид спорта.
Type X	English: Ungrammaticality comes from the fact that the subject pronouns cannot be coindexed with the subject of the sentence.<br><br>Romanian: The ungrammaticality comes from the usage of inappropriate auxiliary verbs.<br><br>Russian: Ungrammaticality case violation.	* Ana broke her arm climbing the tree, didn't they?<br>Ana broke her arm climbing the tree, didn't she?<br><br>* Femeia am adus multe cărţi in bagajele ei grele.<br>Femeia a adus multe cărţi in bagajele ei grele.<br><br>* Ребенок был отправлен новая книга.<br>Ребенку была отправленна новая книга.
Type XI	English: Ungrammaticality comes from wrong word ordering. (Note: All word orderings are possible in Romanian and Russian, just two are possible in English (SOV, OVS).<br><br>Romanian: Ungrammaticality comes from the fact that the sentences contain a copy of	* Are always worried for their children parents.<br>Parents are always worried for their children.<br><br>* Suntem oare şi noi suntem bineveniţi la sărbătoare?

	the aux in its initial position for question formation. It is possible in English, but not Russian.<br><br>Russian: Ungrammaticality comes from the wrong ordering of interrogative pronouns.	Suntem oare şi noi bineveniţi la sărbătoare?<br><br>*Кто-то кого-то узнал но мы не знаем, кого кто.<br>Кто-то кого-то узнал но мы не знаем, кто кого.
Type XII	English: Ungrammaticality comes from the violation of "real-world-knowledge" sentence structure.<br><br>Romanian: Ungrammaticality comes from the missing verb "to have", which needs to be repeated with the auxiliary in negative constructions; note that the verb "to be" does not need to be repeated in such constructions.<br><br>Russian: Ungrammaticality comes from the missing noun after extraction from inside the DP (note that extraction from inside the DP is grammatical in Russian but not in Romanian or English).	* I was surprised for you to get married.<br>I was surprised that you got married.<br><br>* Eu am răspuns la mesaj dar tu nu ai.<br>Eu am răspuns la mesaj dar tu nu ai răspuns.<br><br>Soţia era gata de plecare iar soţul nu era.<br>Soţia era gata de plecare iar soţul nu.<br><br>* Хорошую Александра купила.<br>Хорошую Александра купила книгу.

Table 5. List of ungrammaticality types with examples. Note: the asterisk marks the ungrammatical example of the sentence.

References:

Bird, S., Klein, E., & Loper, E. (2009). *Natural language processing with Python: analyzing text with the natural language toolkit*. " O'Reilly Media, Inc."

Ciochina, L. M., Boyd, V., Ortega, L. S., Malancea-Malac, D., Midrigan, D., & Corina, D. P. (2020). A representative corpus of the romanian language: Resources in underrepresented languages. *LREC 2020 - 12th International Conference on Language Resources and Evaluation, Conference Proceedings*, *May*, 3291–3296.

Harnish, M. J., Beatty, W. W., Nixon, S. J., & Parsons, O. A. (1994). Performance by normal subjects on the shipley institute of living scale. *Journal of Clinical Psychology*, *50*(6), 881–883. https://doi.org/https://doi.org/10.1002/1097-4679(199411)50:6<881::AID-JCLP2270500611>3.0.CO;2-4

Ivanova, I., Salmon, D. P., & Gollan, T. H. (2013). Multilingual naming test in alzheimer's disease: Clues to the origin of naming impairments. *Journal of the International Neuropsychological Society*, *19*(3), 272–283. https://doi.org/10.1017/S1355617712001282

Linebarger, M. C., Schwartz, M. F., & Saffran, E. M. (1983). Sensitivity to grammatical structure in so-called agrammatic aphasics. *Cognition*, *13*(3), 361–392.

Martin, J. D., Blair, G. E., & Vickers, D. M. (1979). Correlation of the quick word test and wide range vocabulary test with the Shipley-Institute of Living Scale. *Educational and Psychological Measurement*, *39*(4), 935–937.

Shipley, W. C. (1940). A Self-Administering Scale for Measuring Intellectual Impairment and Deterioration. *Journal of Psychology: Interdisciplinary and Applied*, *9*(2), 371–377. https://doi.org/10.1080/00223980.1940.9917704
